# Injury patterns and cumulative injury burden among U.S. competitive fencers: A survey

**DOI:** 10.1371/journal.pone.0344263

**Published:** 2026-03-16

**Authors:** Katharine Holmes, Mary Rojas, Periklis Giannakis, Jashvant Poeran, Lindsay Bottoms, Alexis Colvin

**Affiliations:** 1 Department of Medical Education, Icahn School of Medicine at Mount Sinai, New York, New York, United States of America; 2 Department of Anesthesiology, Critical Care and Pain Management, Hospital for Special Surgery, New York, New York, United States of America; 3 Department of Psychology, Sport, and Geography, University of Hertfordshire, Hatfield, United Kingdom; 4 Department of Orthopedics, Icahn School of Medicine at Mount Sinai, New York, New York, United States of America; Universidade de Aveiro Escola Superior de Saude de Aveiro, PORTUGAL

## Abstract

**Background:**

Fencing is a highly asymmetrical sport that combines both repetitive upper-extremity and lower-extremity actions. Although fencing related injuries have been described in clinical- and competition-based cohorts, population level data capturing both training and competition exposures and cumulative injury burden remains limited.

**Objective:**

To characterize injury patterns, mechanisms, and anatomical distribution among adult competitive fencers and to examine associations between training related exposures and reported injury burden.

**Methods:**

Adult competitive fencers registered with USA Fencing were invited to complete an anonymous web-based survey capturing demographics, training and competition exposures, and self-reported fencing related injuries. Injury burden was defined as experiencing three or more lifetime fencing-related injuries among injured respondents. Multivariable logistic regression was used to examine associations between training exposures and injury burden with continuous predictors modeled using restricted (natural) cubic splines to allow for non-linear relationships. Descriptive analyses, correlation analyses, and Poisson regression were performed as sensitivity analyses.

**Results:**

Among 303 respondents, 270 (89.1%) reported at least one fencing related injury, accounting for 571 total injuries. Overuse injuries predominated and most frequently involved the knee, ankle and dominant upper extremity with gradual-onset, non-contact mechanisms accounting for the majority of the injuries. Upper-extremity injuries were significantly more likely to occur on the dominant side. In multivariable analyses, years of fencing experience demonstrated a significant non-linear association with higher injury burden, while weekly training volume showed a non-linear association that approached statistical significance. Age at starting fencing, competition frequency, and sex were not independently associated with injury burden. Sensitivity analyses using Poisson regression yielded qualitatively similar findings.

**Conclusions:**

Among adult competitive fencers, higher injury burden is most strongly associated with cumulative training exposure, particularly years of fencing experience, with additional contribution from weekly training volume. Injury patterns are characterized by overuse and pronounced dominant-side upper-extremity involvement, consistent with the sport’s asymmetrical biomechanical demands. These findings underscore the importance of monitoring cumulative exposure and addressing asymmetrical loading to mitigate recurrent injury burden in fencing.

## Introduction

Fencing is a combat sport characterized by rapid acceleration and decelerations, repetitive unilateral upper extremity movements, and asymmetrical lower extremity loading. These biomechanical demands create a unique injury profile that includes both acute traumatic injuries and chronic overuse conditions. As participation in competitive fencing spans a wide age range and often extends over decades, understanding how cumulative exposure related to injury burden is particularly important.

Prior studies of fencing injuries have largely focused on elite competition settings, emergency department presentations, or clinic-based cohorts which may overrepresent acute or medically attended injuries while under capturing training related and overuse conditions [1–6]. Emergency department surveillance studies, such as that by Stanicki et al. [7], have characterized acute injuries requiring urgent medical care, while clinical series have provided insight into injuries presenting for specialty evaluation. More recently, Cross et al. [8] reported a predominance of lower extremity injuries and a clear vulnerability of the dominant side in a clinical population. Similarly, a retrospective analysis of French elite fencers by Gondouin et al. [9] demonstrated a high overall injury burden with a predominance of lower extremity injuries, while also showing that nearly all upper extremity injuries involved the dominant weapon arm, highlighting the role of repetitive, asymmetric loading in this sport. Systematic reviews have likewise identified the ankle and knee as the most commonly reported sites of fencing-related injury [10].

Several studies focusing on competition-related injuries have similarly identified strains and sprains of the knee and ankle as the most common injury types [1,11]. An informal survey conducted by *American Fencing* magazine in 1992 likewise reported the knee and ankle as the most frequently injured body parts [12]. Although these studies contributed to early characterization of fencing injury patterns, they were largely limited to competition-based or clinical populations. Consequently, large-scale, population-based data capturing injuries sustained during both practice and competition and detailing injury mechanisms, anatomic distribution, and injury burden, particularly with respect to dominant versus non-dominant side involvement, remain limited. In addition, medically attended injury datasets may not capture chronic or recurrent conditions that do not prompt clinical evaluations, limiting insight into cumulative injury burden among long-term participants.

Reports of upper-extremity injuries in fencing, especially overuse conditions affecting the dominant weapon arm, have varied across study designs and populations. The highly unilateral nature of fencing, requiring repeated weapon handling, grip force, wrist extension, and forearm rotation, suggests that dominant-side upper-extremity injuries may be an important, but under characterized, component of fencing-related injury patterns. However, population-based data describing injury type, anatomical distribution, laterality, and mechanism across training and competition settings remain limited.

Understanding how training- related exposures contribute to injury burden presents additional challenges. Prior work suggests that greater training volume, longer participation history, and higher competitive level may be associated with increased injury risk, yet the form of these relationships is not well defined. Many studies have relied on categorization of continuous exposure variables, which may obscure a nonlinear dose-response patterns and limit interpretability. Analytic approaches that allow for flexible modeling of exposure-injury relations are needed to better characterize how cumulative and intensity related factors contribute to recurrent injury.

Accordingly, the objectives of this study were to: (1) describe the prevalence, mechanism and anatomic distribution, and laterality of fencing related injuries in a population based cohort of competitive fencers and (2) examine associations between training related exposures and reported injury burden. We hypothesize that greater cumulative exposure, reflected by training volume and years of fencing experience, would be associated with higher injury burden. To address potential nonlinear relationships, we modeled continuous exposure variables using restricted cubic splines within a multivariable framework.

## Methods

### Study design and participants

This cross-sectional, population-based survey study was conducted among adult competitive members of USA Fencing. Institutional Review Board approval was obtained from the Icahn School of Medicine at Mount Sinai (Protocol 24–00128). All participants provided informed consent electronically before completing the survey, which included an introductory statement explaining study purpose, voluntary participation, and data anonymity.

Fencers aged 18 years or older listed as “Competitive Members” within USA Fencing’s database (N = 14,839 members) as of January 1, 2024 were invited to participate in a web-based platform survey (SurveyMonkey Inc. San Mateo, CA), with reminder emails sent at one-month intervals over a four-month recruitment period (January – April 2024).

### Survey instrument and data collection

The survey instrument consisted of 79 items and was developed by KH, a three-time U.S. Olympic fencer, in consultation with medical experts and the USA Fencing Sports Medicine Director (see Supplementary Materials 1). Prior to dissemination, the survey instrument underwent pilot testing for content clarity and face validity. Independent review by four additional competitive fencers (two U.S. Olympic fencers and two U.S. National Team members), who provided feedback on question clarity, relevance to fencing-specific injury experiences, and interpretability. Minor revisions were made based on this feedback to improve wording and clarity. However, psychometric testing was not done.

The survey collected information on demographics (age, gender, ethnicity, hand dominance), training and competition exposure, and self-reported fencing related injuries. Participants reported their total lifetime number of fencing injuries using a capped response format (0–5); those reporting more than five injuries were instructed to provide detailed information for the five most impactful injuries. For each injury, respondents selected the injury type from a predefined list (e.g., ligament tear/rupture, tendon tear/rupture, tendonitis/tenosynovitis, fracture, meniscal tear, contusion, puncture/laceration), with an option to specify “other” if applicable, and reported the anatomical location, side, mechanism, setting (training or competition), time loss, treatment, and degree of recovery.

### Injury definitions and outcome measures

A broad injury definition was intentionally used to capture both acute and overuse conditions occurring in fencing training and competition settings, recognizing that many fencing-related injuries do not result in formal medical evaluation or time-loss but nonetheless affect athlete participation. For descriptive analyses, participants were categorized as having no reported injuries, one to two injuries, or three or more injuries.

For multivariable modeling, injury burden was operationalized as a binary outcome defined as experiencing three or more lifetime fencing related injuries with one to two injuries among injured fences. Participants reporting no injuries were excluded from the logistic regression analyses to focus on factors associated with a higher injury burden among injured fencers and to avoid sparse outcome categories given the small number of self-reported injured respondents.

### Statistical analysis

Descriptive statistics were reported as medians and interquartile ranges for continuous variables and as counts and percentages for categorical variables. Group comparisons across injury burden categories were performed using chi-square tests for categorical variables and Kruskal–Wallis tests for continuous variables, as appropriate.

Spearman correlation coefficients were calculated to examine bivariate association between injury count and exposure variables including years of fencing, weekly training volume, and competition frequency. These analyses were conducted to inform the multivariable modeling strategy.

Multivariable logistic regression was used to examine associations between training-related factors and reported injury burden. Continuous covariates, including age started fencing, years of fencing experience, total weekly training hours, and number of competitions, were modeled using restricted (natural) cubic splines to allow for nonlinear associations. Knots were placed at the 10th, 50th, and 90th percentiles of each continuous predictor. Gender was included as a binary covariate. Overall effects of spline terms were assessed using Wald Tests.

As a sensitivity analysis, Poisson regression models were fit using categorized versions of the exposure variables with injury count as the outcome. Because respondents were limited in reporting details for their five more severe injuries, injury counts reflect a truncated measure of burden thus Poisson models were used for comparison purposes only. Results of categorical logistic and Poisson models are presented in the Supplementary Materials.

Statistical significance was set at p < 0.05, and all analyses were conducted using IBM SPSS Statistics (version 28; IBM Corp., Armonk, NY) and SAS software (version 9; SAS Institute Inc., Cary, NC).

## Results

### Participant characteristics and injury burden

Of 303 survey respondents, 270 (89.1%) reported at least one fencing-related injury and were included in descriptive analyses. Injury burden was categorized as no injuries, one to two injuries, or three or more injuries. Demographic characteristics were generally similar across injury burden groups (Table 1), with no significant differences in age or sex distribution.

**Table 1:** Characteristics of study sample by injury burden (n = 303).

Characteristic	No Injury Group (n=33)	1-2 injury (n=138)	≥3 Injuries (n=132)	χ² (df) / Kruskal–Wallis	P value
**Age, median (Q1-Q3)**	44.0 (24.5, 64.0)	46.5 (32.0, 60.2)	54.0 (34.0, 64.0)	2.98	0.236
**Age started fencing, median (Q1-Q3)**	23.0 (18.0, 49.5)	18.5 (13.0, 38.0)	18.0 (13.0, 31.8)	8.93	0.012
**Gender**				2.8 (2)	0.246
Male, % (n)	75.8 (25)	63.0 (87)	67.7 (93)		
Female, % (n)	24.2 (8)	37.0 (51)	29.5 (39)		
**Level of Competition**				28.91. (4)	< .001
Below National, % (n)	58.6 (17)	20.3 (26)	15.6 (17)		
National, % (n)	31.0 (9)	70.3 (90)	66.1 (72)		
International, % (n)	10.3 (3)	9.4 (12)	18.3 (20)		
**Hand**				1.91 (2)	0.389
Left	24.2 (8)	15.2 (21)	20.0 (26)		
Right	75.8 (25)	84.8 (117)	80.0 (104)		
**Years Fencing, median (Q1-Q3)^1^**	7.0 (3.0,19.0)	13.0 (6.0, 23.0)	18.5 (10.0, 28.0)	18.75	< .001
**Training Hours/Week, median (Q1-Q3)^1^**	10.0 (7.8, 14.0)	11.0 (7.0, 15.0)	13.0 (8.0, 17.0)	6.06	0.048
**Number of Competitions**	4.0 (2.5, 7.0)	6.0 (3.0, 10.0)	6.0 (4.0, 11.25)	9.27	0.01

^1^Q1-Q3 first and third quartile

In contrast, several indicators of cumulative exposure differed significantly by injury burden. Fencers with three or more injuries reported greater years of fencing experience, higher weekly training volume, greater competition participation, and higher competitive level compared with those reporting fewer or no injuries (Table 1).

### Injury patterns, location, side and mechanism

Across the 571 reported injuries, overuse conditions, particularly tendonitis/tenosynovitis (26.4%), were the most frequent injury types (Table 2). Injuries most frequently involved the knee (18.2%) and ankle (11.7%), as well as the upper extremity, with a predominance of dominant-side involvement for upper-limb injuries. Non-contact and gradual-onset mechanisms accounted for the majority of injuries, and most injuries occurred during training rather than competition.

**Table 2:** Most common injuries and body part affected, side of injury, and mechanism (N = 571).

10 most common injuries	N	%
*Tendonitis/Tenosynovitis*	151	26.4
*Ligament tear/rupture*	86	15.1
*Fracture*	42	7.4
*Contusion*	40	7
*Tendon tear/rupture*	37	6.5
*Puncture/laceration/cut*	31	5.4
*Meniscal tear*	26	4.6
*Strain*	22	3.9
*Muscle tear*	20	3.5
**10 most common body part injured**		
*Knee*	104	18.2
*Ankle*	67	11.7
*Elbow*	61	10.7
*Finger*	41	7.2
*Shoulder*	37	6.5
*Wrist*	29	5.1
*Hip*	28	4.9
*Hand*	27	4.7
*Foot*	27	4.7
*Hamstring*	20	3.5
**Side of injury**		
*Dominant side*	349	61.1
*Non dominant side*	170	29.8
*Center*	44	7.7
**Mechanism**		
*Overuse*	253	44.3
*Acute action/accident during bout*	137	24
*Direct hit from opponent*	79	13.8
*Fall*	33	5.8
*Collision with opponent*	30	5.3

Counts are not displayed for responses of “Other” nor is missing data.

“Other” responses were coded where possible based on what was specified.

Upper-extremity injuries were significantly more likely to involve the dominant side compared to non-upper extremity injuries (χ² = 54.3, p < 0.001) whereas lower-extremity and trunk injuries were more evenly distributed. Detailed distributions of injury type, anatomical location, laterality, and mechanism are presented in Table 2.

### Associations between exposure variables and injury count

Spearman correlation analyses demonstrated positive associations between injury count and cumulative exposure measures, including years of fencing (ρ = 0.30, p < 0.001) and weekly training hours (ρ = 0.13, p = 0.03; see Table 3). These bivariate relationships informed the multivariable modeling strategy.

**Table 3:** Spearman correlation between training factors and injury (N = 303).

Variable	Age	Age Started	Years Fencing	Competitions per Year	Training Hours per Week
Age Started	0.51**	--			
Years Fencing	0.54**	−0.21**	--		
Competitions per Year	−0.16**	−0.1	−0.04	--	
Training Hours per Week	−0.12*	−0.16**	−0.15*	0.23**	--
Injury Count	0.14*	−0.09	0.30**	0.14*	0.13*

**. Correlation is significant at the 0.01 level (2-tailed).

*. Correlation is significant at the 0.05 level (2-tailed).

Sample size varied from 265–303.

### Multivariable analyses of injury burden

Among injured fencers, multivariable logistic regression modeling the odds of experiencing three or more injuries demonstrated a statistically significant improvement in fit compared with an intercept-only model (see Table 4). Years of fencing experience showed a significant non-linear association with injury burden (overall Wald p = 0.002), with increasing odds of multiple injuries across the observed range. Weekly training volume demonstrated a non-linear association that approached statistical significance (overall Wald p = 0.06), with higher injury burden observed at moderate-to-high training levels and no further increase at the highest volumes. Age at starting fencing, number of competitions, and gender were not independently associated with higher injury burden after adjustment.

**Table 4:** Multivariable logistic regression modeling the odds of experiencing three or more injuries (N = 263).

Independent Variable	Modeling approach	DF	Overall P-value
Age started fencing	Restricted cubic spline	2	0.86
Total training hours	Restricted cubic spline	2	0.06
Number of competitions	Restricted cubic spline	2	0.08
Years fencing	Restricted cubic spline	2	0.002
Gender (male vs female)	Binary indicator	1	0.26

Continuous predictors were modeled using restricted (natural) cubic splines with knots at the 10th, 50th, and 90th percentiles. P-values correspond to Type 3 Wald tests assessing the overall association of each predictor with injury burden.

As a sensitivity analysis, Poisson regression models using categorized predictors and injury count as the outcome yielded qualitatively similar findings. Years of fencing remained the most consistent predictor of injury outcomes, while associations with training volume were attenuated in the count-based models. Results of these categorical logistic and Poisson analyses are provided in Supplementary Materials S2 and S3 in S1 File.

### Predicted probabilities of injury burden

**Fig 1:**
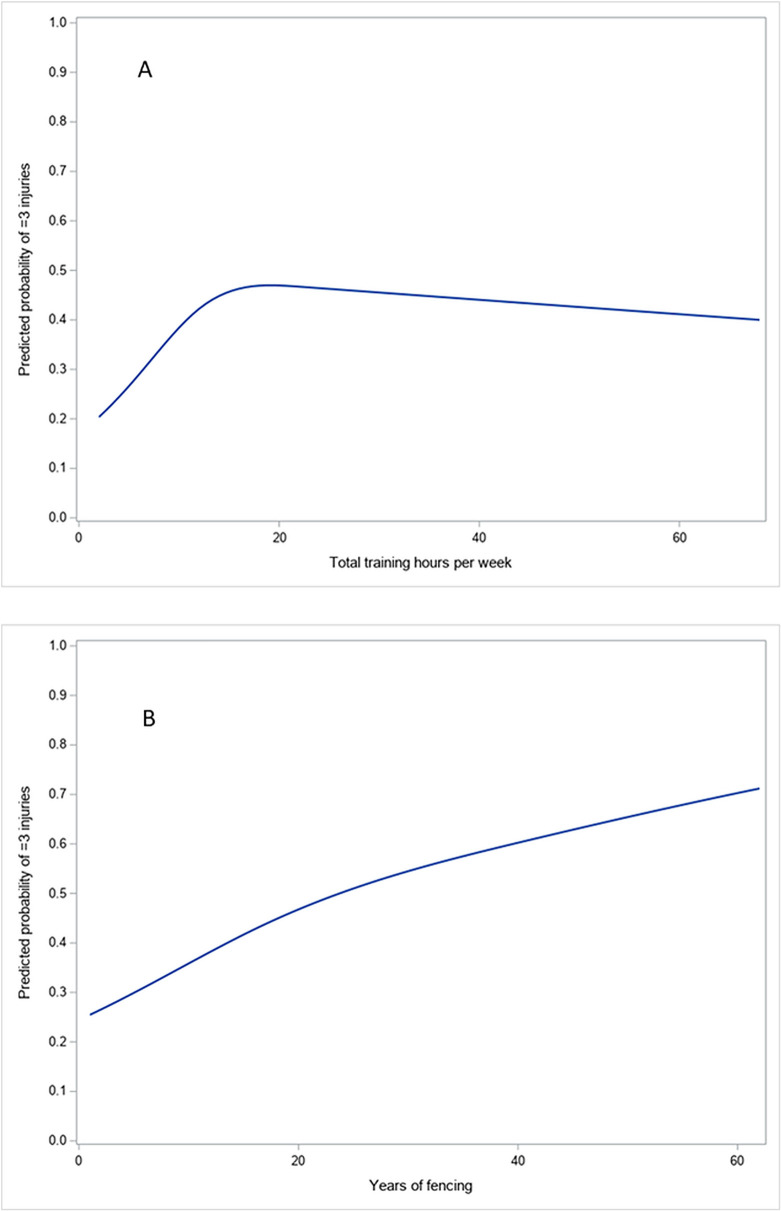
Predicted probability of experiencing three or more injuries by training exposure. **(A)** Adjusted predicted probability of experiencing three or more injuries across total training hours. **(B)** Adjusted predicted probability of experiencing three or more injuries across years of fencing. Predictions were estimated from multivariable logistic regression models using restricted cubic splines. Curves are shown separately by gender (coded 0 and 1), with all other covariates held at their median values. The smooth curves illustrate non-linear associations between cumulative training exposure and injury burden.

To aid interpretation of the nonlinear models, predicted probabilities of experiencing three or more injuries were plotted across the observed ranges of weekly training hours and years of fencing (Fig 1). The predicted probability of higher injury burden increased steadily with years of fencing, consistent with cumulative exposure. In contrast, training volume demonstrated a nonlinear pattern, with highest predicted probabilities observed at moderate-to-high volumes and no further increase at the highest reported volumes.

## Discussion

In this cross-sectional survey of competitive fencers, injury burden was most strongly associated with cumulative training exposure, particularly years of fencing experience, with additional contribution from weekly training volume. Across analytic approaches, including bivariate correlations, spline-based regression, and Poisson regression sensitivity analysis, years of fencing consistently emerged as the most robust covariate of higher injury burden. This pattern supports an interpretation centered on cumulative exposure accumulates over time, rather than demographic characteristics or competition participation alone.

Descriptive analyses of 571 reported injuries provide important clinical context for these associations. Overuse injuries, particularly tendonitis and tenosynovitis, were the most common injury types, and injuries most frequently involved the knee, ankle, and dominant upper extremity. The predominance of gradual-onset and non-contact mechanisms highlights the contribution of repetitive loading rather than acute trauma. These findings align with the mechanical demands of fencing, which combine repeated unilateral upper-extremity actions with high- frequency lower-extremity loading during footwork and lunging.

The pronounced asymmetry observed in upper-extremity injuries, with a strong predominance on the dominant side, is clinically and biomechanically plausible. These findings both align with and extend prior clinic- and competition-based studies of fencing injuries. Similar to the clinical cohort reported by Cross et al. [8], we observed a predominance of lower extremity injuries, with the knee being the most frequently affected joint, along with clear dominance-side vulnerability. However, important differences emerge due to study design and population. Cross et al. examined injuries presenting to sports medicine and orthopedic clinics, capturing clinically evaluated cases that likely represent more acute or medically attended injuries. In contrast, the present population-based survey captured reported injuries occurring during both practice and competition, including overuse conditions such as tendonitis and tenosynovitis that may not prompt formal medical evaluation. Accordingly, we observed a higher proportion of dominant-side upper extremity overuse injuries, particularly involving the elbow, shoulder, and finger, which are likely underrepresented in clinic-based cohorts.

These findings are further supported by recent studies in elite fencing populations. A retrospective analysis of French elite fencers by Gondouin et al. [9] demonstrated a high overall injury burden with a predominance of lower extremity injuries, while also showing that nearly all upper extremity injuries involved the dominant weapon arm. Similarly, Thompson et al. [13], examining injuries among U.S. National Team and Olympic fencers, reported frequent lower extremity injuries, particularly involving the knee, along with marked laterality consistent with fencing’s asymmetric loading patterns. Together, these studies reinforce the biological plausibility of our observed injury distribution while highlighting that population-based surveys capture a broader spectrum of cumulative and overuse injuries beyond those presenting for clinical care.

The anatomical distribution of injuries observed in the present study reflects the asymmetrical biomechanical demands of fencing. Lower extremity injuries were most common at the knee and ankle, likely related to repetitive lunging, deceleration, and rapid directional changes intrinsic to fencing footwork. In contrast, upper extremity injuries clustered at the elbow, finger, and shoulder, consistent with repetitive weapon handling, gripping, and striking motions. The strong predominance of dominant-side upper extremity injuries further underscores the role of unilateral loading and repetitive stress in injury development. While lower extremity injuries were common, they were more bilaterally distributed, consistent with alternating propulsion, lunging, and recovery movements inherent to fencing footwork.

Compared with emergency department–based surveillance, such as the NEISS analysis by Stanicki et al. [7], our findings provide complementary insight into chronic and cumulative injury patterns across a wider range of fencing participation contexts. While Stanicki et al. similarly reported lower extremity injuries as most common, emergency-based surveillance inherently excludes overuse injuries and training-related conditions that do not result in emergency care. Likewise, the systematic review by Swatowska et al. [10] identified ankle sprains and lower-limb strains as the most frequently reported fencing injuries across heterogeneous study designs, while emphasizing substantial variability in injury definitions, settings, and athlete populations. Our findings build on this literature by characterizing reported injury burden across both training and competition settings and by explicitly examining cumulative exposure as a key determinant of recurrent injury.

The nonlinear association between training volume and injury burden observed in spline-based models further underscores the importance of flexible analytic approaches. Injury burden increased with training volume at lower to moderate levels, but did not rise uniformly at the highest training volumes. This pattern may reflect a combination of training adaptation, selective retention of more resilient athletes at higher levels of exposure or exposure modification following injury. Such dynamics could be obscured by linear or categorical models and highlight the value of spline-based methods for examining dose response relationships in sports injury research.

Notably, competition frequency was not independently associated with higher injury burden after adjustment, despite showing a positive bivariate correlation with injury count. This finding suggests that training exposure may contribute more substantially to cumulative injury burden than competition exposure alone, particularly for overuse conditions. Similarly, age at starting fencing and gender were not independently associated with higher injury burden in multivariable analyses indicating that exposure related factors outweigh demographic characteristics once participation history is accounted for.

With respect to generalizability, the sex and weapon distribution of the study sample appears broadly representative of adult competitive fencing in the United States. Epee was the most commonly represented weapon, followed by foil and saber, and male fencers comprised approximately two-thirds of participants across weapons, consistent with available USA fencing membership summaries (provided to the authors). Although the median age of respondents was relatively high, multivariable analyses indicated that cumulative fencing exposure rather than chronological age was most strongly associated with injury burden. Accordingly, these findings are most applicable to adult competitive fencers with extended participation histories and may not directly generalize to youth or early-career athletes.

## Limitations

Several limitations should be considered. First, the response rate was low (2%), introducing a substantial risk of selection bias and limiting external validity. Although this response rate is comparable to that reported in other USA Fencing surveys [14], it does not mitigate the possibility of non-response bias. Respondents may differ systematically from non-respondents, particularly with respect to injury history, engagement with the sport, and training exposure. The low response rate also limits external validity and generalizability. As a result, absolute injury prevalence and injury counts in this sample are likely overestimated relative to the broader competitive fencing population. However, the primary aim of this study was to characterize injury patterns and examine associations between cumulative exposure and injury burden rather than to generate precise prevalence estimates. While selection bias may distort absolute injury frequencies, the observed relationships between training exposure and injury are internally consistent, biologically plausible, and aligned with prior clinical and epidemiologic studies of fencing. Accordingly, the results should be viewed as exploratory and hypothesis-generating, intended to inform future prospective surveillance rather than definitive risk estimates.

Additional limitations include the cross-sectional design, which precludes causal inference, and injury histories were self-reported and limited to the five most severe injuries, resulting in truncated counts. This constraint likely contributed to underdispersion in count-based models and motivated the use of injury burden as clinically meaningful outcome. Exposure measures were also self-reported and may be subject to recall bias. Despite these limitations, the consistency of findings across descriptive analyses, correlation analyses, spline-based logistic regression and Poisson sensitivity models support the robustness of the observed associations.

Taken together, these findings emphasize the clinical relevance of cumulative exposure and asymmetrical loading in fencing-related injuries. Monitoring training volume, promoting balanced strength and conditioning, and incorporating recovery strategies may be particularly important for mitigating recurrent injury burden among long-term participants. Future prospective studies incorporating longitudinal exposure tracking and standardized injury surveillance are needed to better define temporal relationships and inform targeted, evidence-based injury prevention strategies in fencing.

## Conclusion

In this cohort of adult competitive fencers, higher injury burden was most strongly associated with cumulative training exposure, particularly years of fencing experience with additional contribution from weekly training volume. Descriptive analyses demonstrated that overuse injuries predominated and most frequently involved the knee, ankle, and dominant upper extremity, with pronounced asymmetry favoring dominant-side upper-limb injuries. Together, these findings suggest the recurrent fencing related injury reflects sustained, asymmetrical mechanical loading accumulated over time rather than isolated acute events.

The nonlinear relationships observed between training exposure and injury burden highlight the importance of monitoring cumulative load and considering adaptation and recovery in long-term participants. These results emphasize the potential value in training loan management, balance strength, conditioning, and recovery strategies in mitigating recurrent injury burden among adult competitive fencers. Future prospective studies incorporating longitudinal exposure tracking and injury surveillance are needed to better define temporal relationships and inform targeted injury prevention strategies.

## Supporting information

S1 File Supplemental materials. As a sensitivity analysis, we conducted additional multivariable regression analyses using categorized versions of the exposure variables to evaluate the robustness of the primary findings. These analyses were performed using both logistic regression, modeling injury burden, and Poisson regression, modeling injury count, with identical sets of categorized predictors included in each model. For the logistic regression analysis, injury burden was defined as experiencing three or more fencing-related injuries compared with one to two injuries among injured fencers. Age at starting fencing, weekly training volume, years of fencing experience, number of competitions, and sex were included as categorical predictors (S1 Table). For the Poisson regression analysis, the total number of fencing-related injuries was modeled as a count outcome using the same categorized predictors. Because respondents were asked to report details for only their five most severe injuries, injury counts represent a truncated measure of burden. As such, Poisson models were used for comparison purposes only and are presented as a sensitivity analysis (S2 Table). Across both modeling approaches, years of fencing experience consistently demonstrated the strongest association with injury outcomes, supporting a cumulative exposure effect. Weekly training volume showed a consistent direction of association across models but was attenuated in the Poisson analysis. Age at starting fencing and sex were not independently associated with injury outcomes in either model. Overall, these supplementary analyses support the robustness of the primary spline-based logistic regression findings presented in the main manuscript.
(DOCX)

S1 Table Logistic regression of injury burden (≥3 injuries vs. 1–2 injuries) using categorized covariates (N = 263). Multivariable logistic regression modeling injury burden among injured fencers, defined as experiencing three or more fencing-related injuries compared with one to two injuries. Age at starting fencing, weekly training volume, years of fencing experience, number of competitions, and sex were included as categorical covariates. Results are presented as adjusted odds ratios (OR) with 95% confidence intervals. This analysis is presented as a sensitivity analysis to the primary spline-based models reported in the main manuscript. Uninjured respondents were excluded from this analysis.
(DOCX)

S2 Table Poisson regression of injury count using categorized covariates (N = 263). Multivariable Poisson regression modeling the total number of fencing-related injuries using the same categorized predictors as in S1 Table. Results are presented as incidence rate ratios (IRR) with 95% confidence intervals. Because respondents were asked to report details for only their five most severe injuries, injury counts represent a truncated measure of burden. This analysis is presented for comparison purposes only. Uninjured respondents were excluded from this analysis.
(DOCX)

S3 File Survey. (DOCX)
