# Supplementary material for: Injury patterns and cumulative injury burden among U.S. competitive fencers: A survey
Source: PLoS One. 2026 Mar 16;21(3):e0344263. doi: 10.1371/journal.pone.0344263 (PMC12991207; doi:10.1371/journal.pone.0344263)
Supplement: S1 File — As a sensitivity analysis, we conducted additional multivariable regression analyses using categorized versions of the exposure variables to evaluate the robustness of the primary findings. These analyses were performed using both logistic regression, modeling injury burden, and Poisson regression, modeling injury count, with identical sets of categorized predictors included in each model. For the logistic regression analysis, injury burden was defined as experiencing three or more fencing-related injuries compared with one to two injuries among injured fencers. Age at starting fencing, weekly training volume, years of fencing experience, number of competitions, and sex were included as categorical predictors (S1 Table). For the Poisson regression analysis, the total number of fencing-related injuries was modeled as a count outcome using the same categorized predictors. Because respondents were asked to report details for only their five most severe injuries, injury counts represent a truncated measure of burden. As such, Poisson models were used for comparison purposes only and are presented as a sensitivity analysis (S2 Table). Across both modeling approaches, years of fencing experience consistently demonstrated the strongest association with injury outcomes, supporting a cumulative exposure effect. Weekly training volume showed a consistent direction of association across models but was attenuated in the Poisson analysis. Age at starting fencing and sex were not independently associated with injury outcomes in either model. Overall, these supplementary analyses support the robustness of the primary spline-based logistic regression findings presented in the main manuscript. (DOCX) [file pone.0344263.s001.docx]

S1 Survey


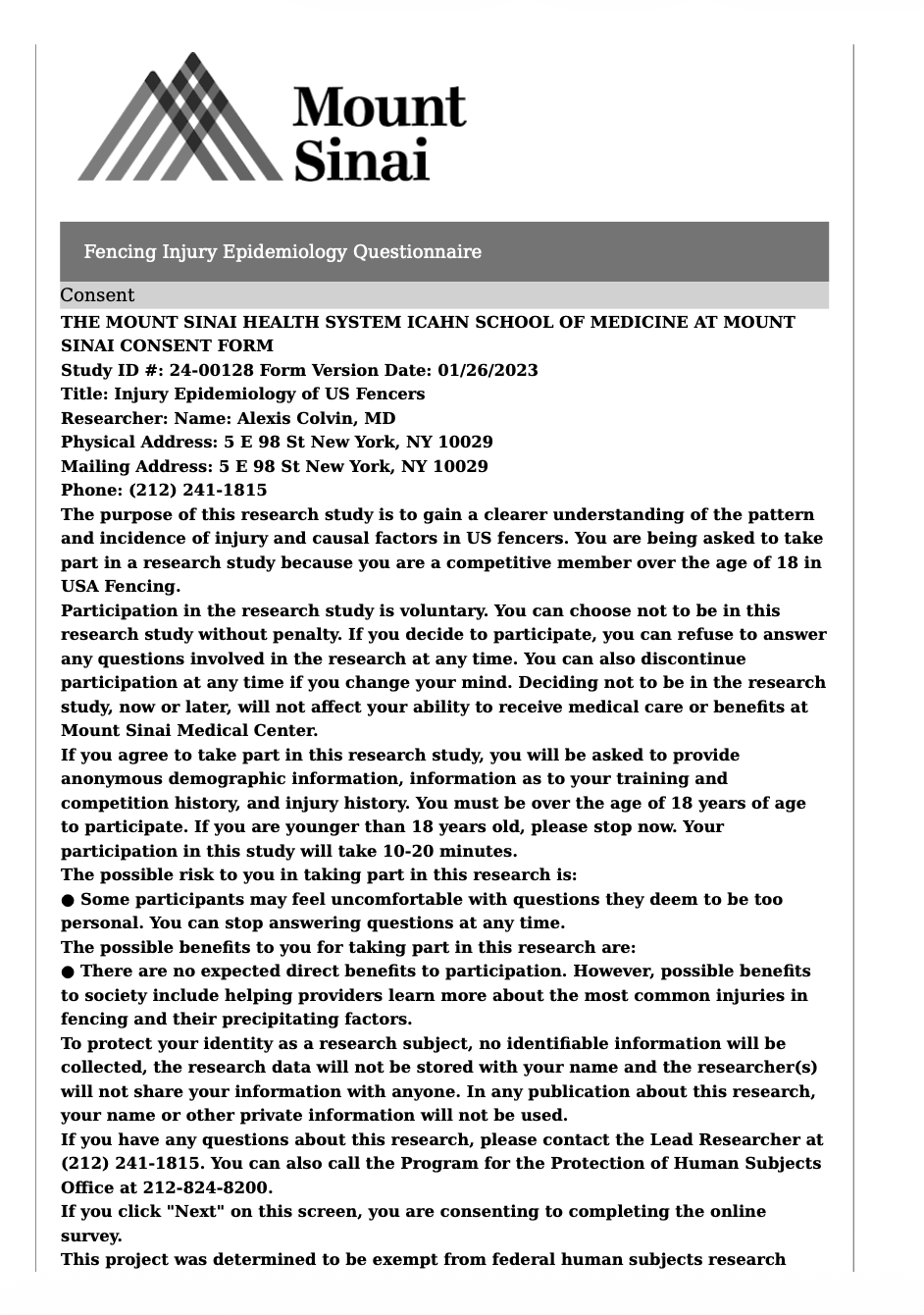


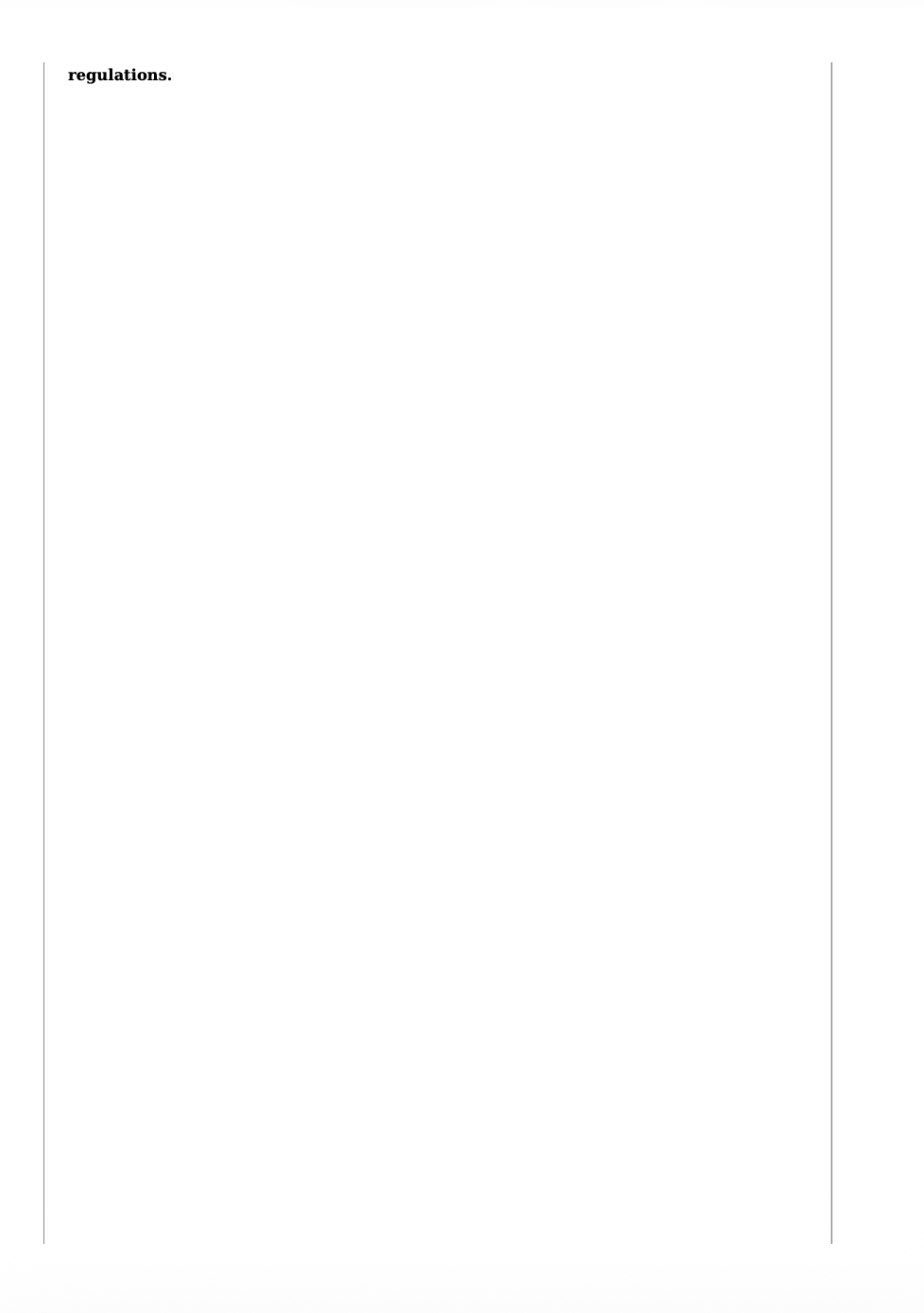


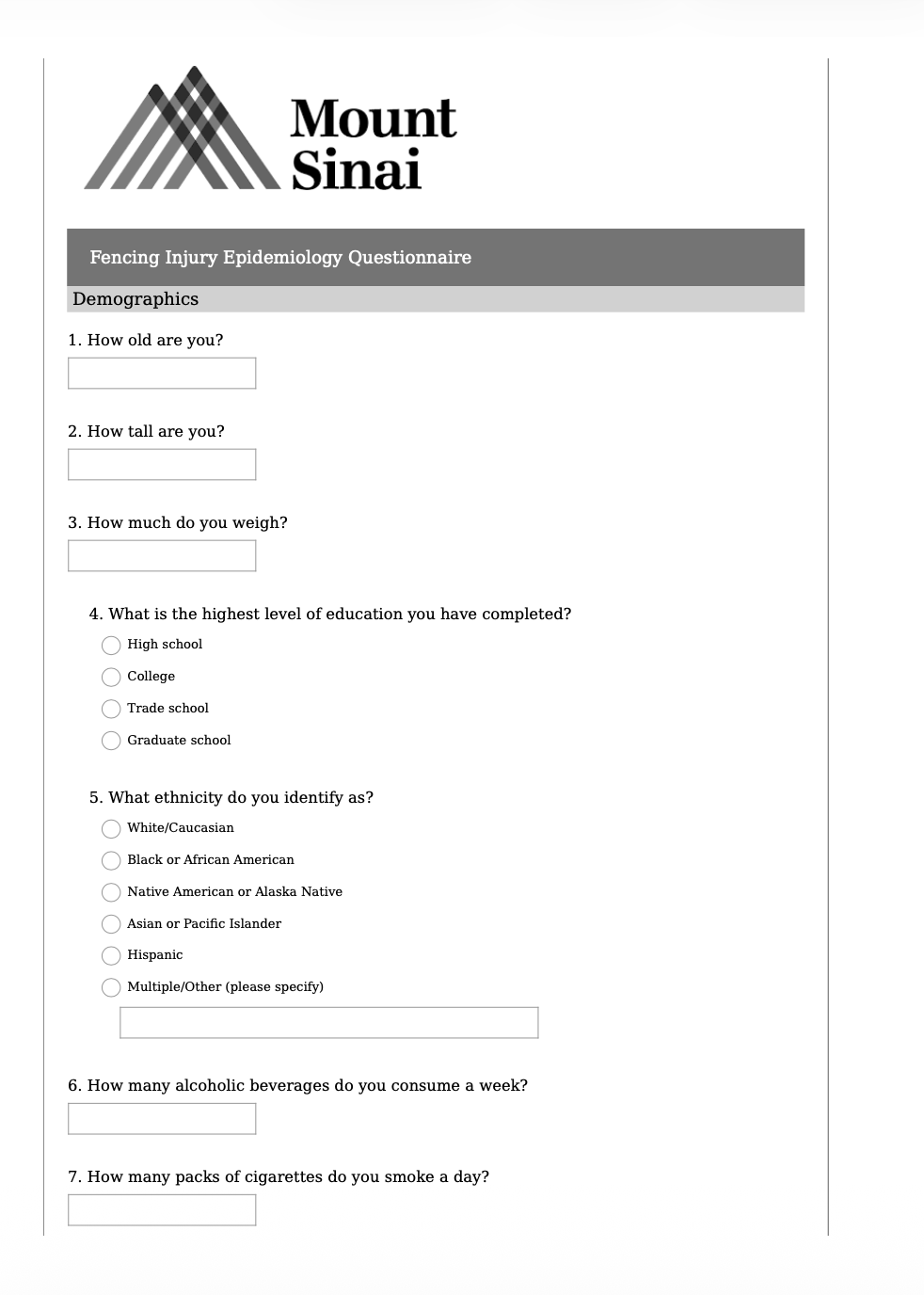


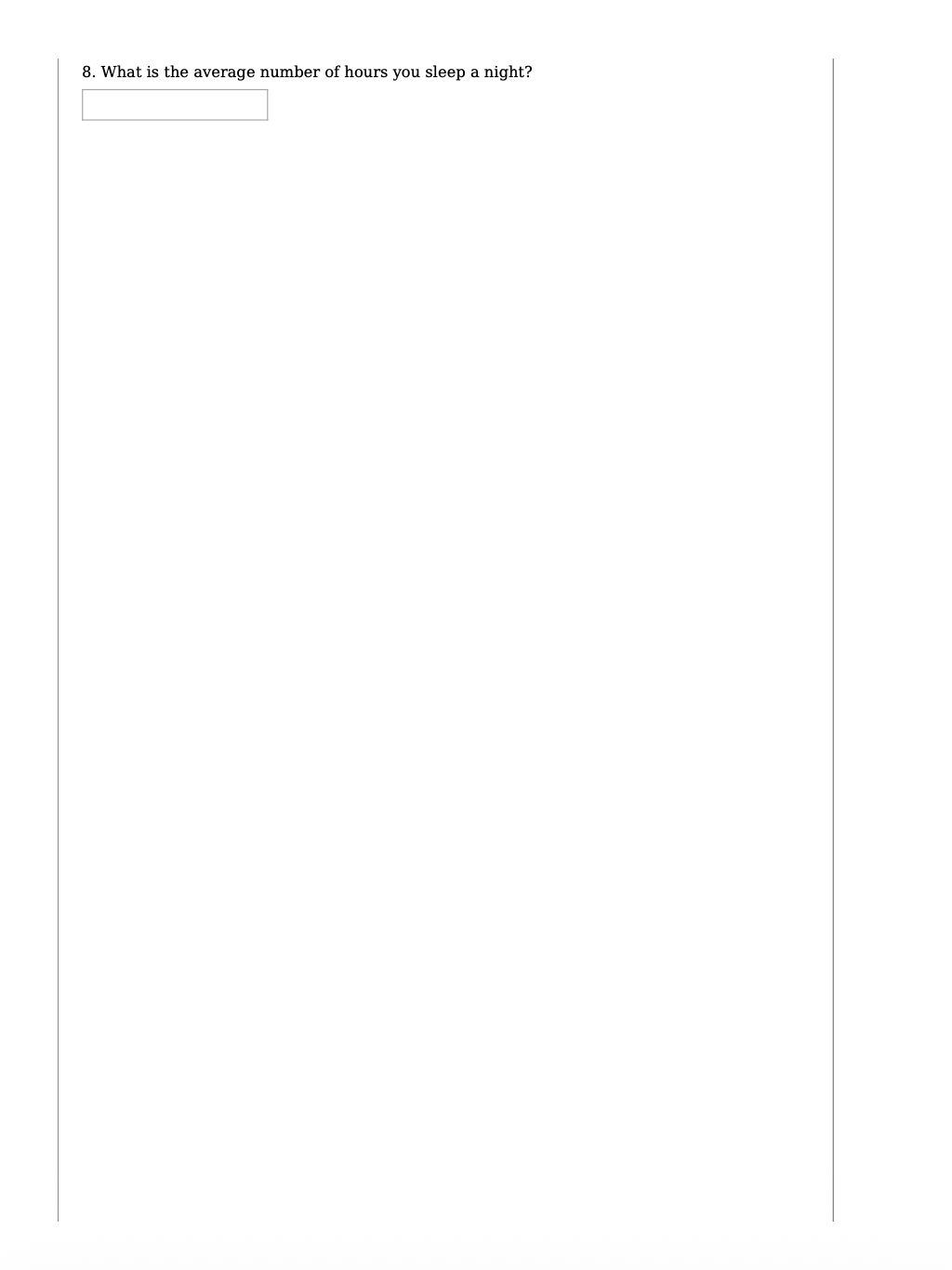


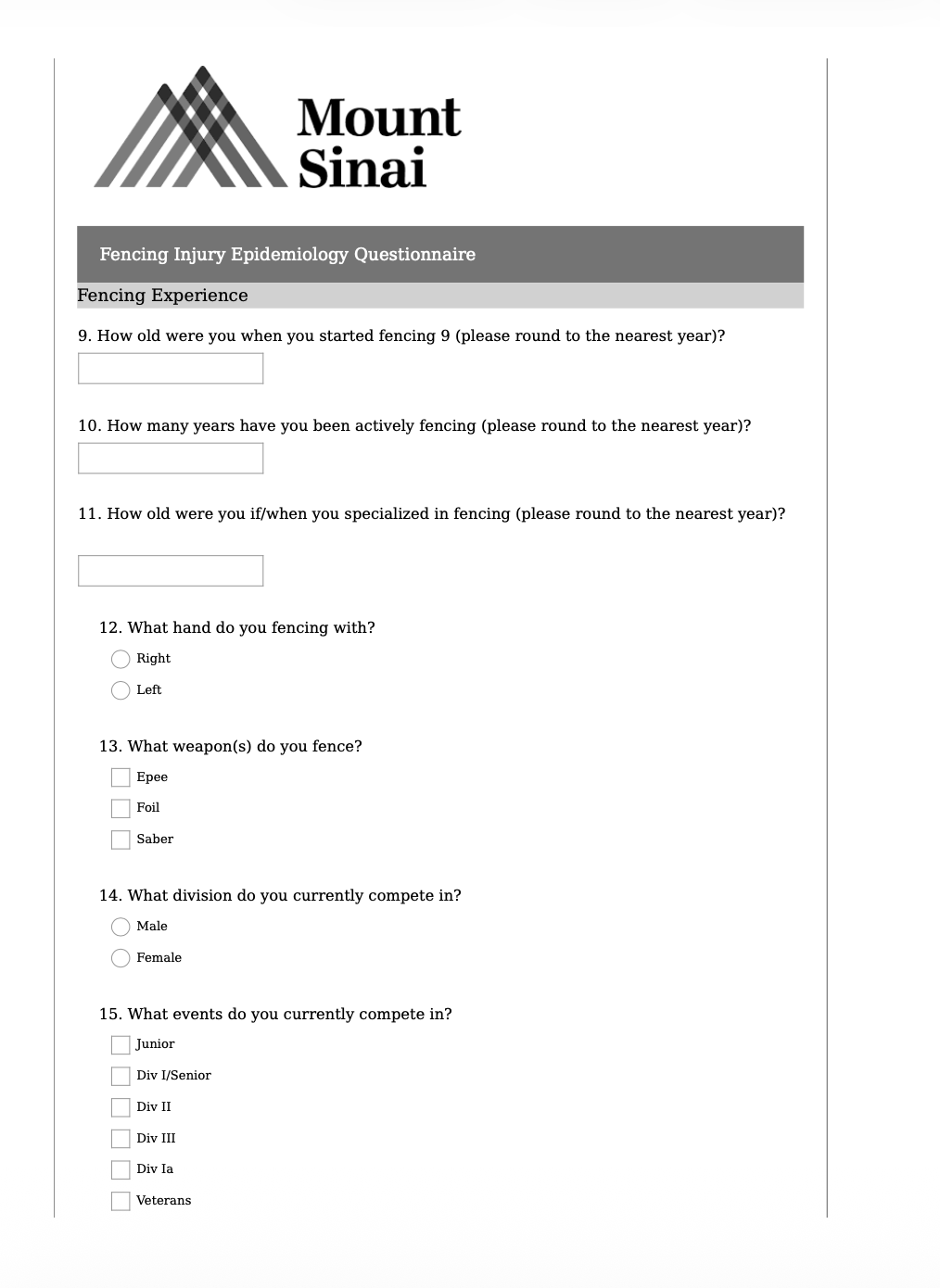


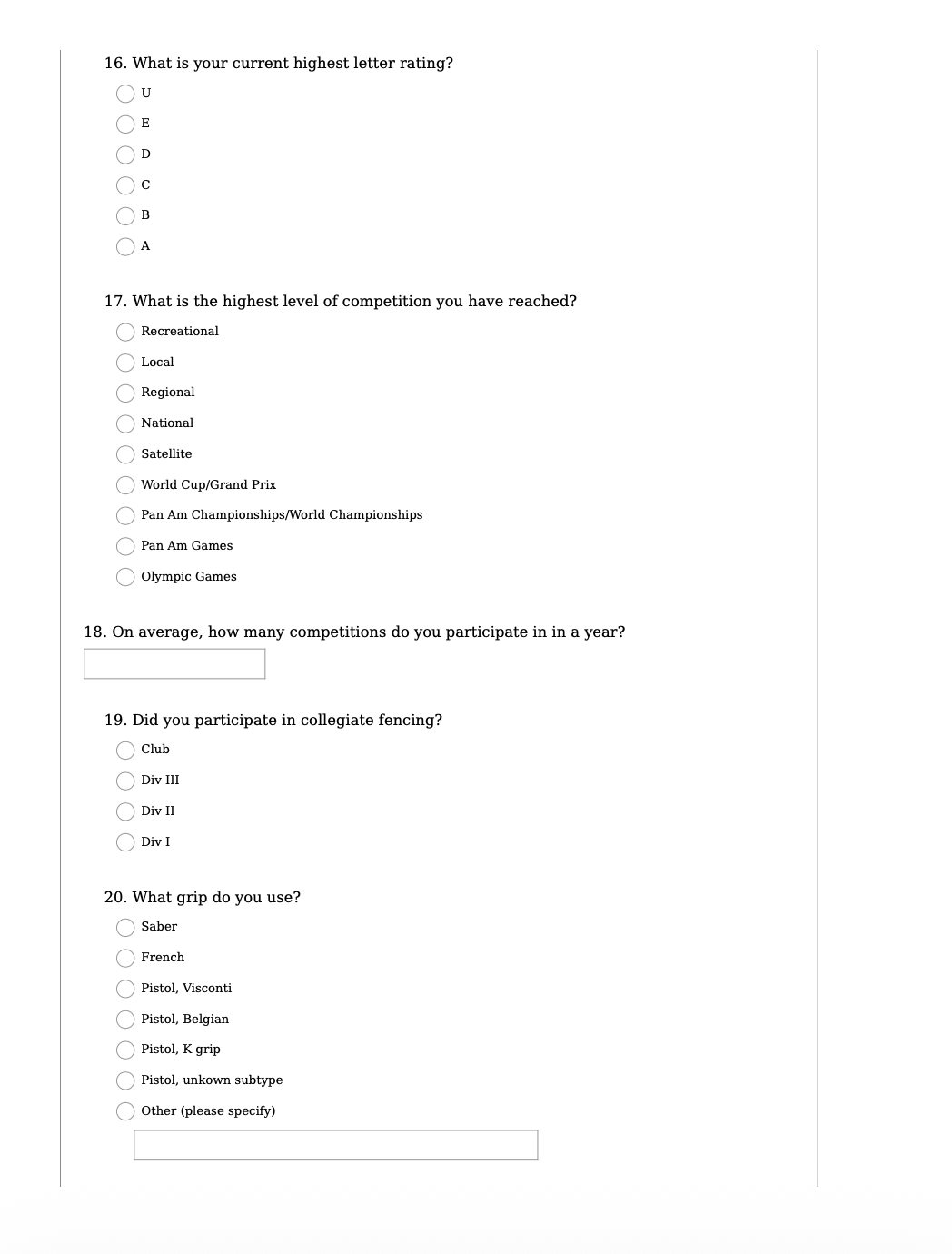


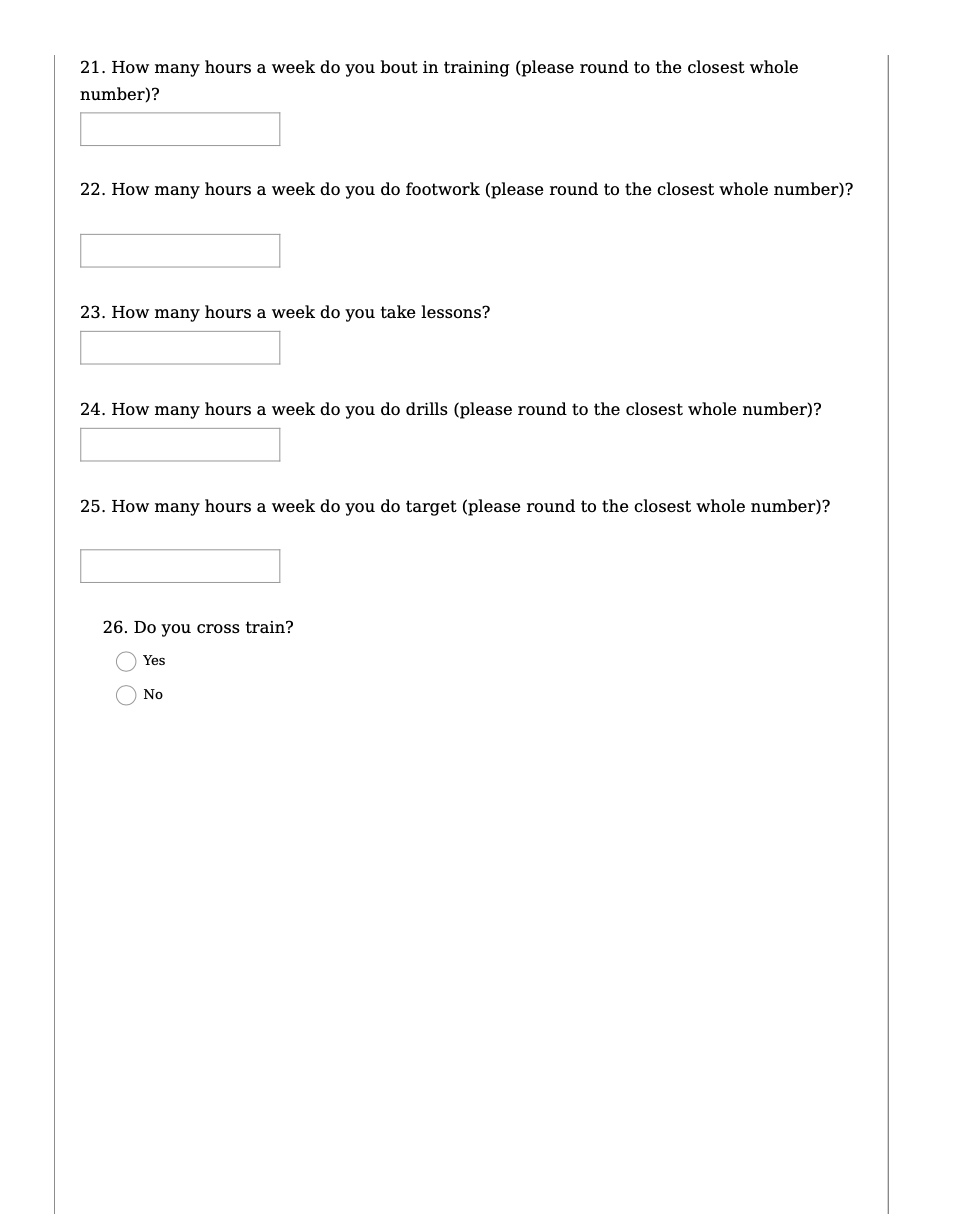


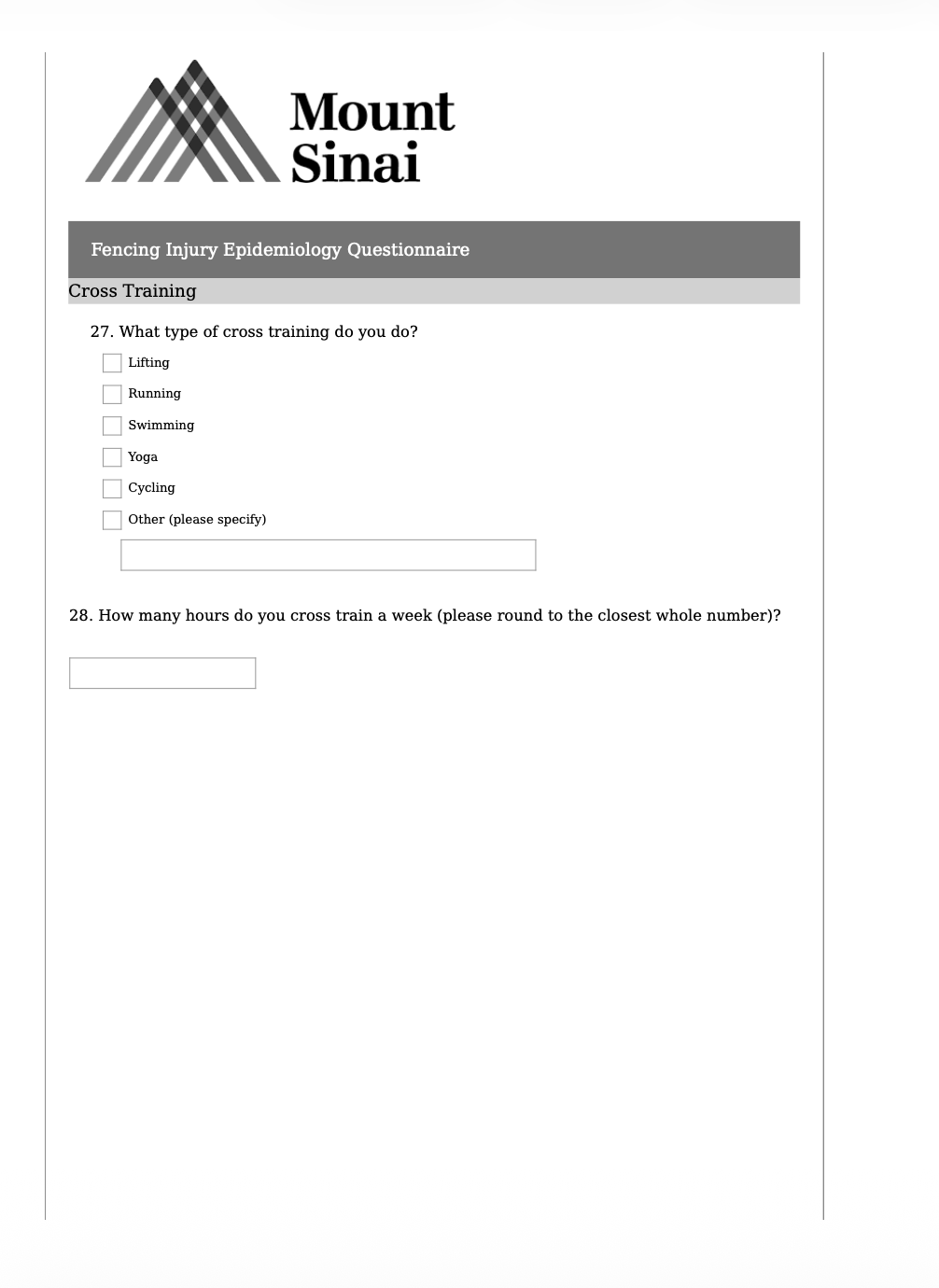


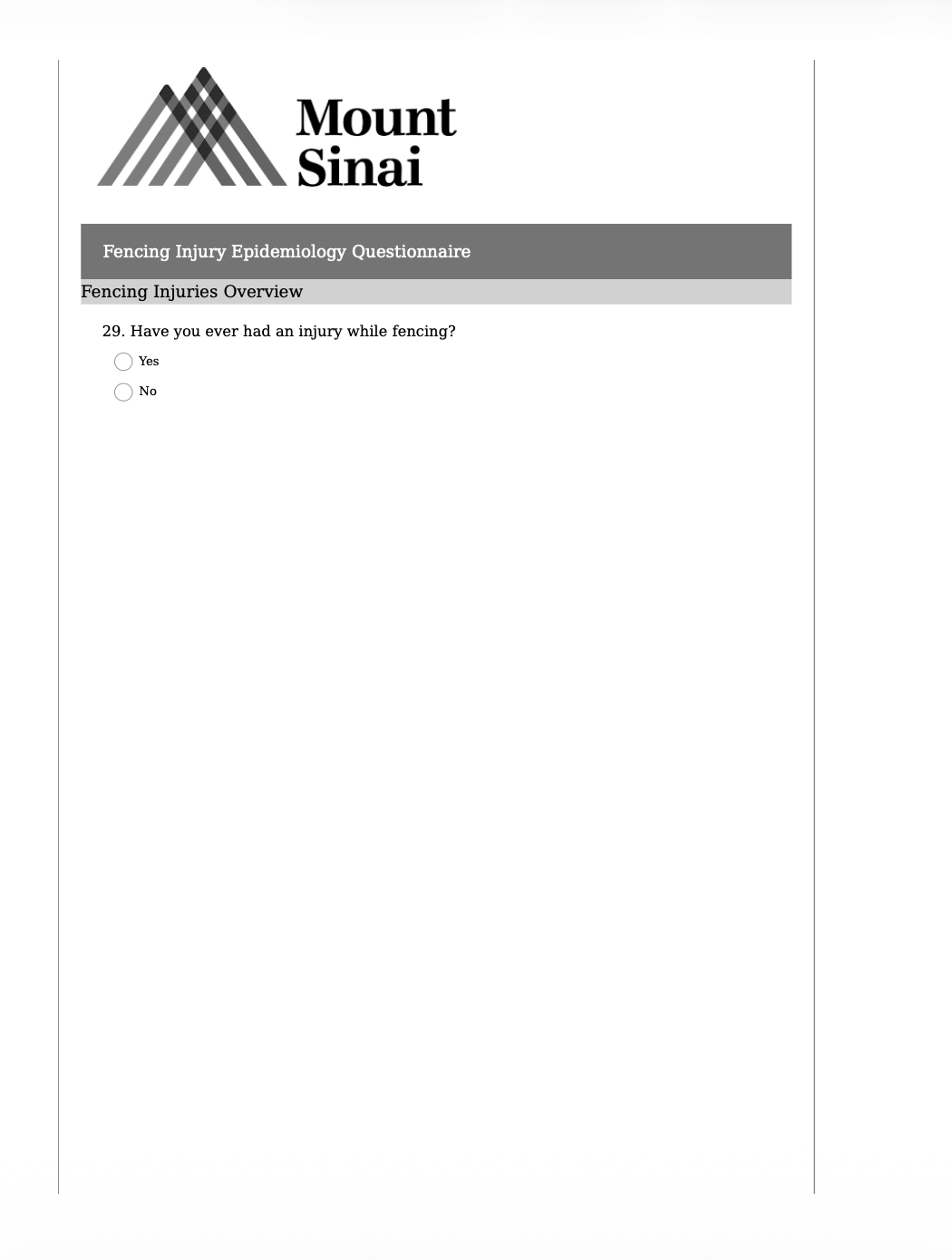


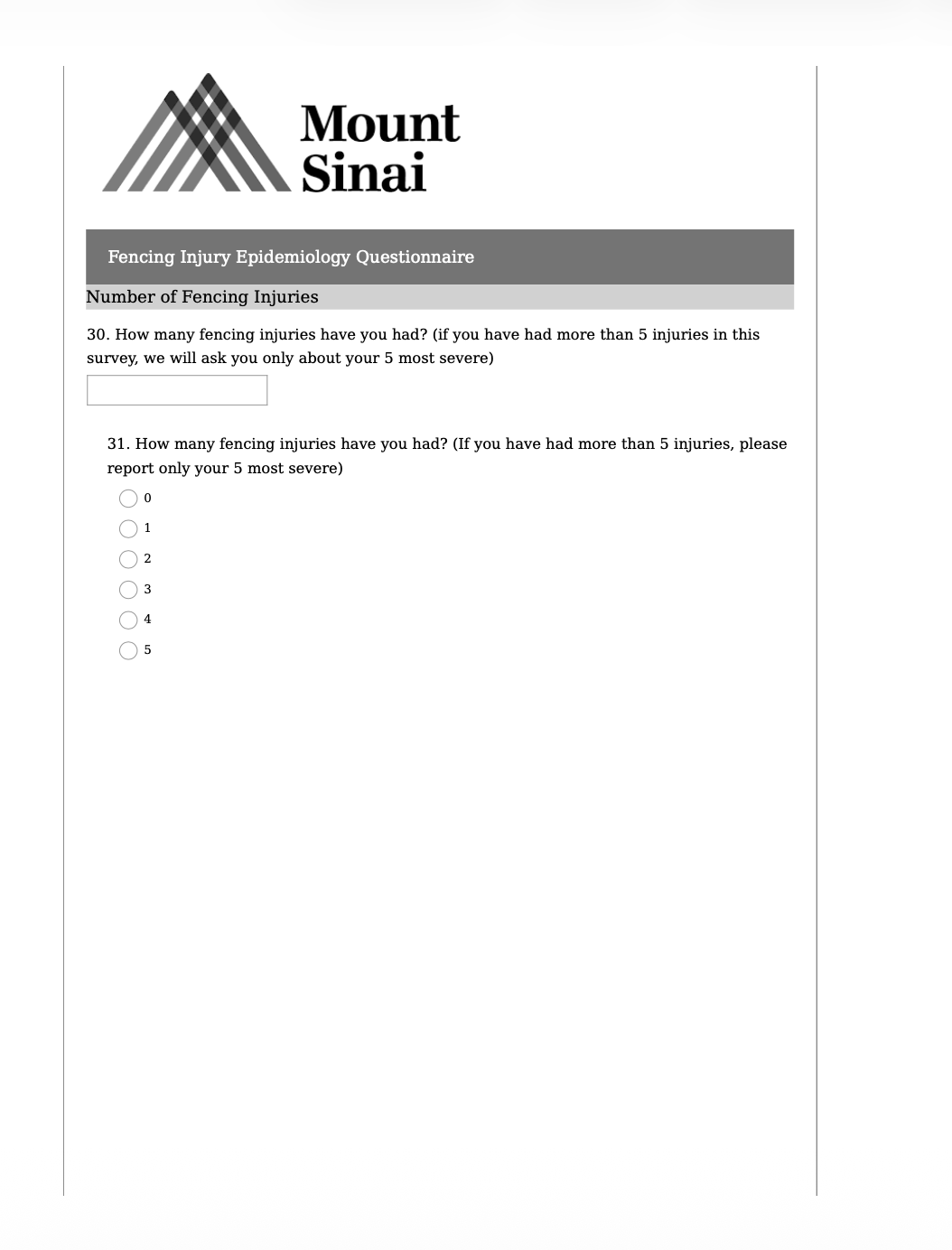


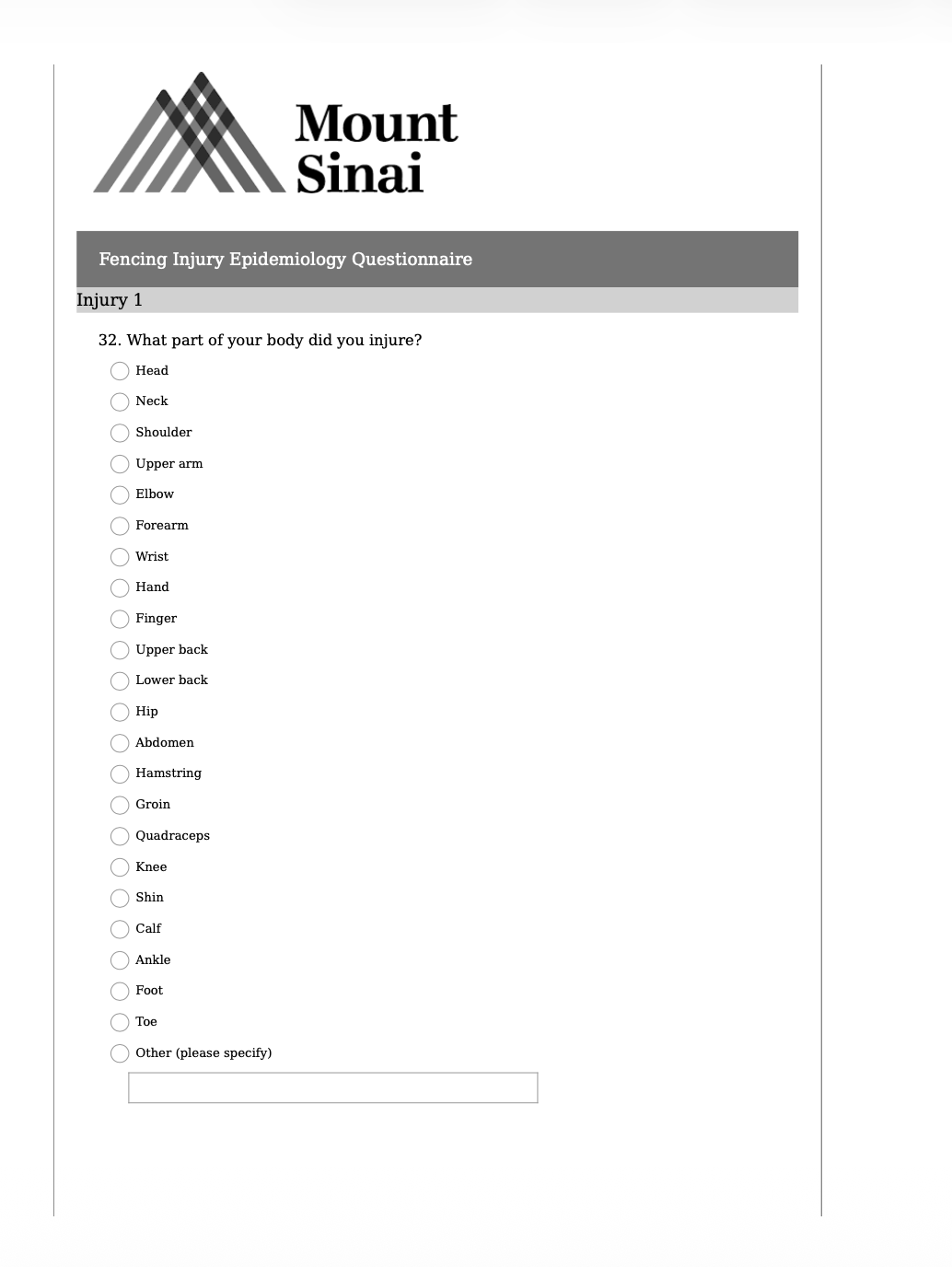


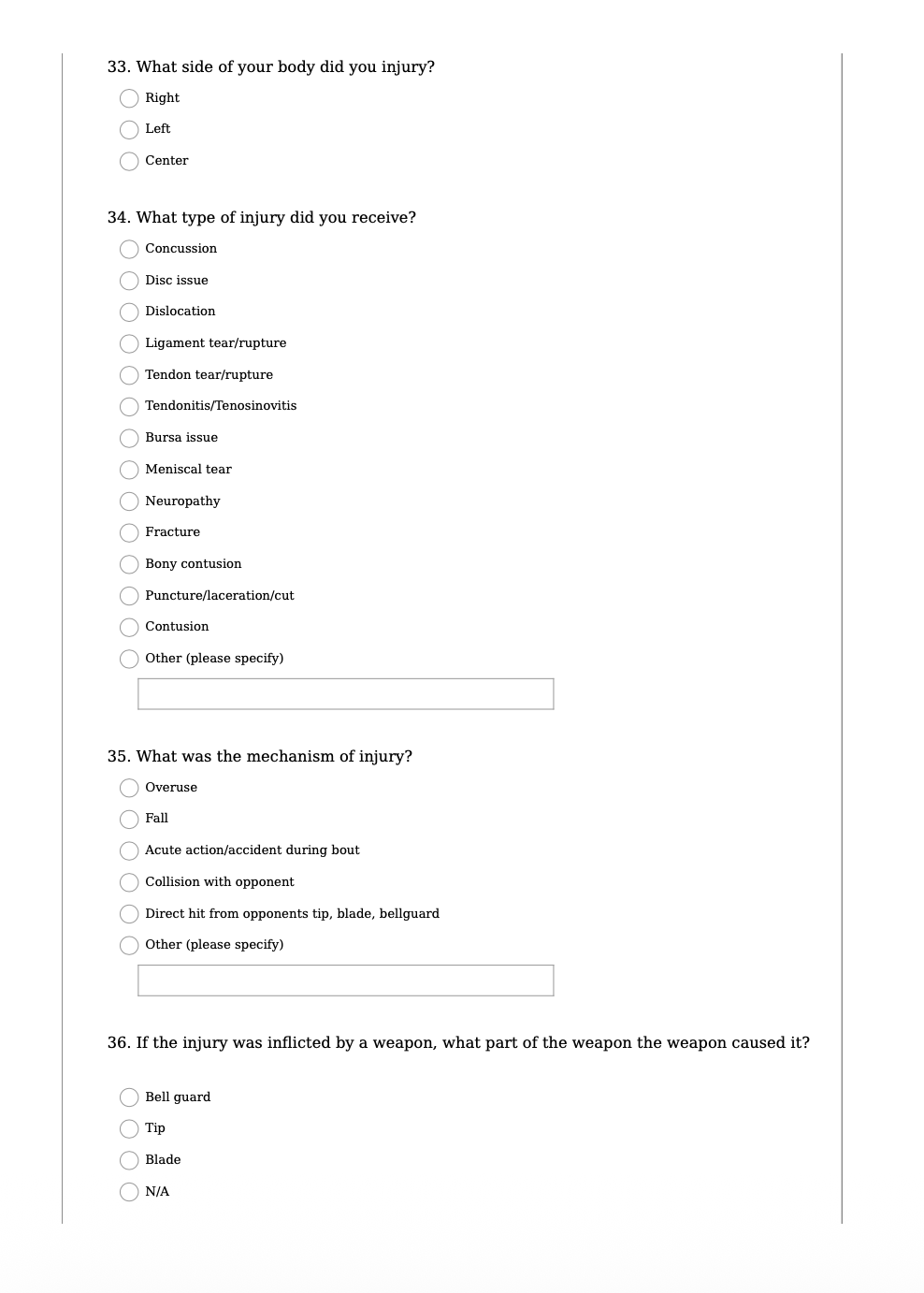


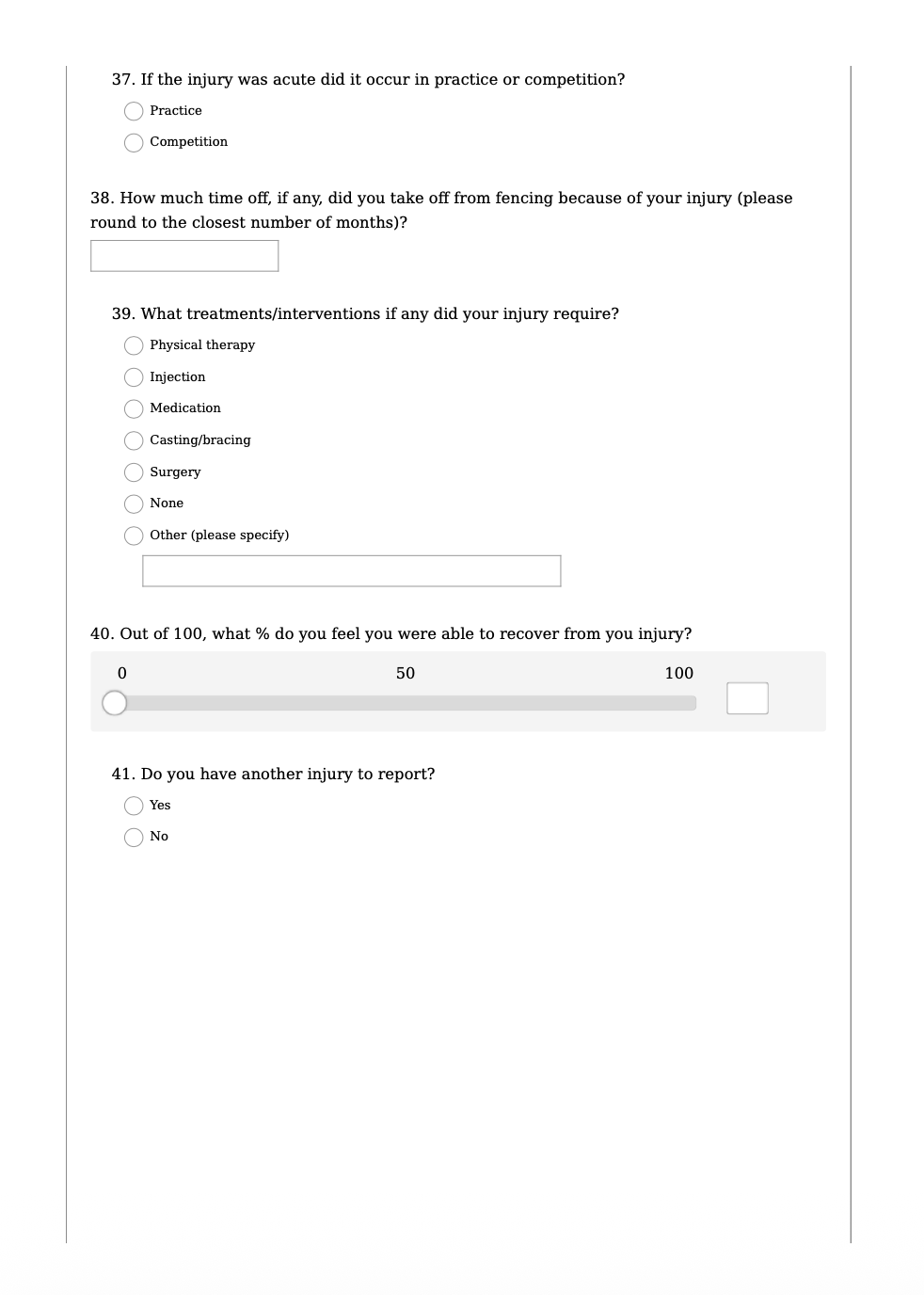


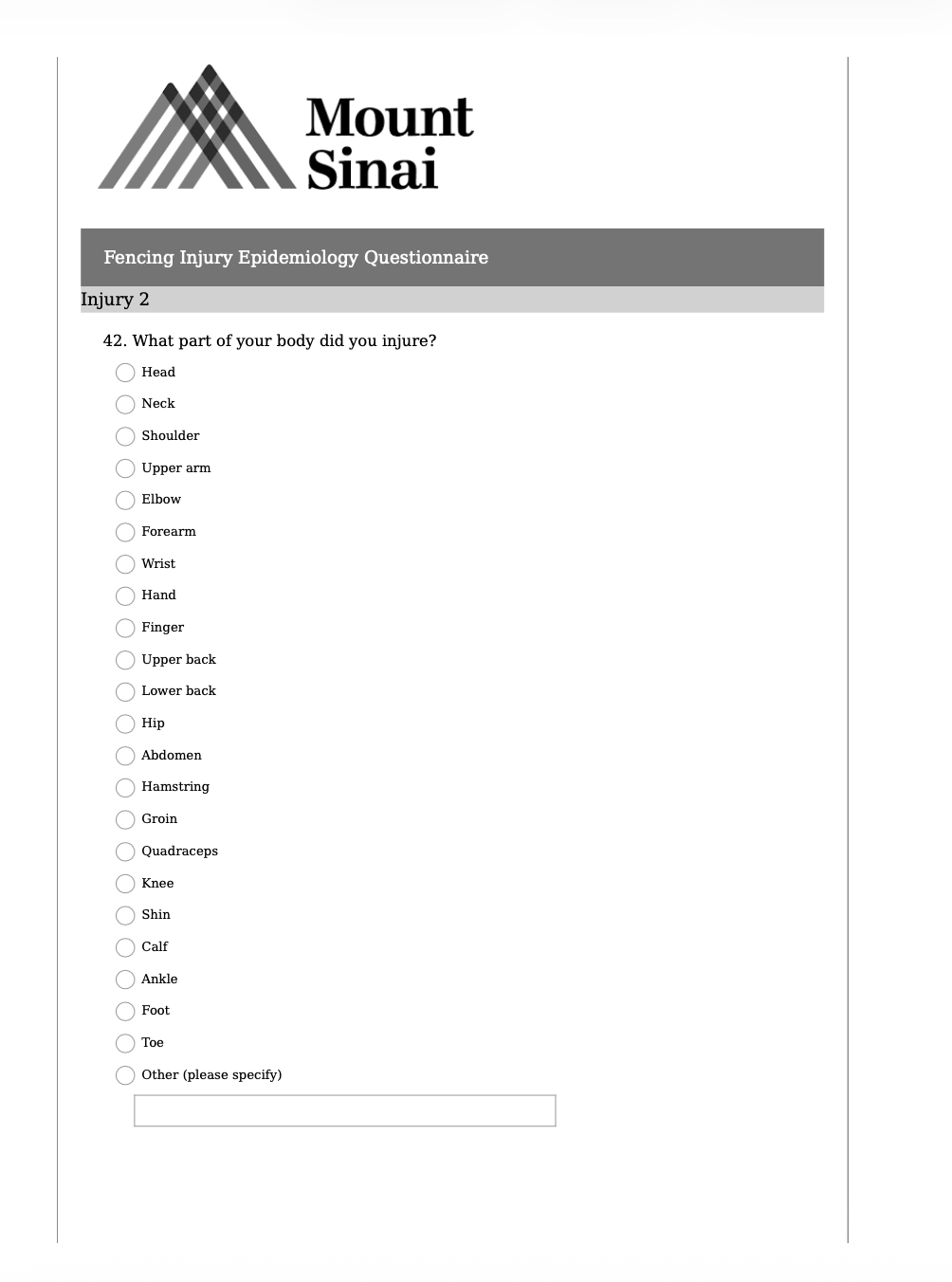


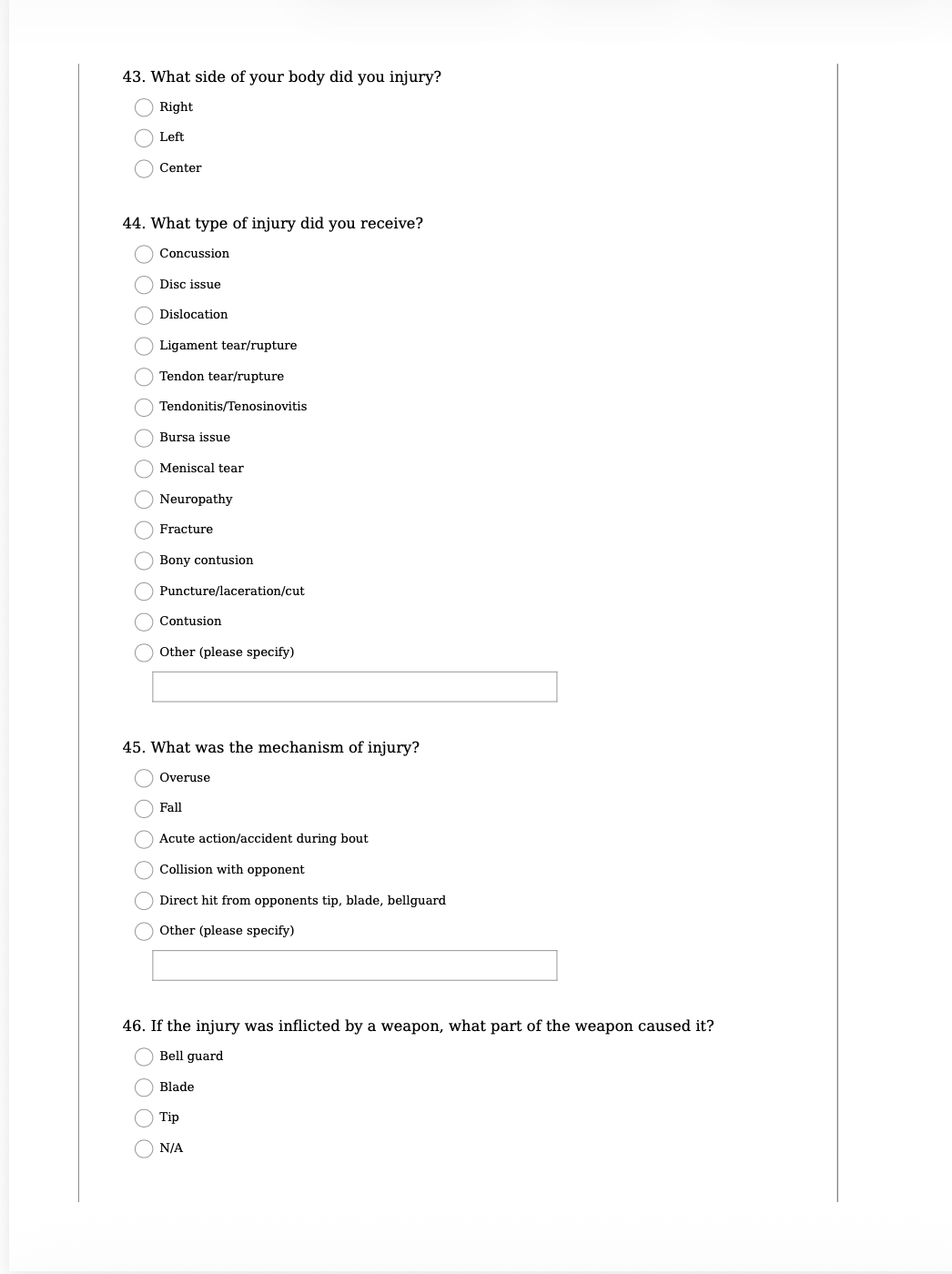


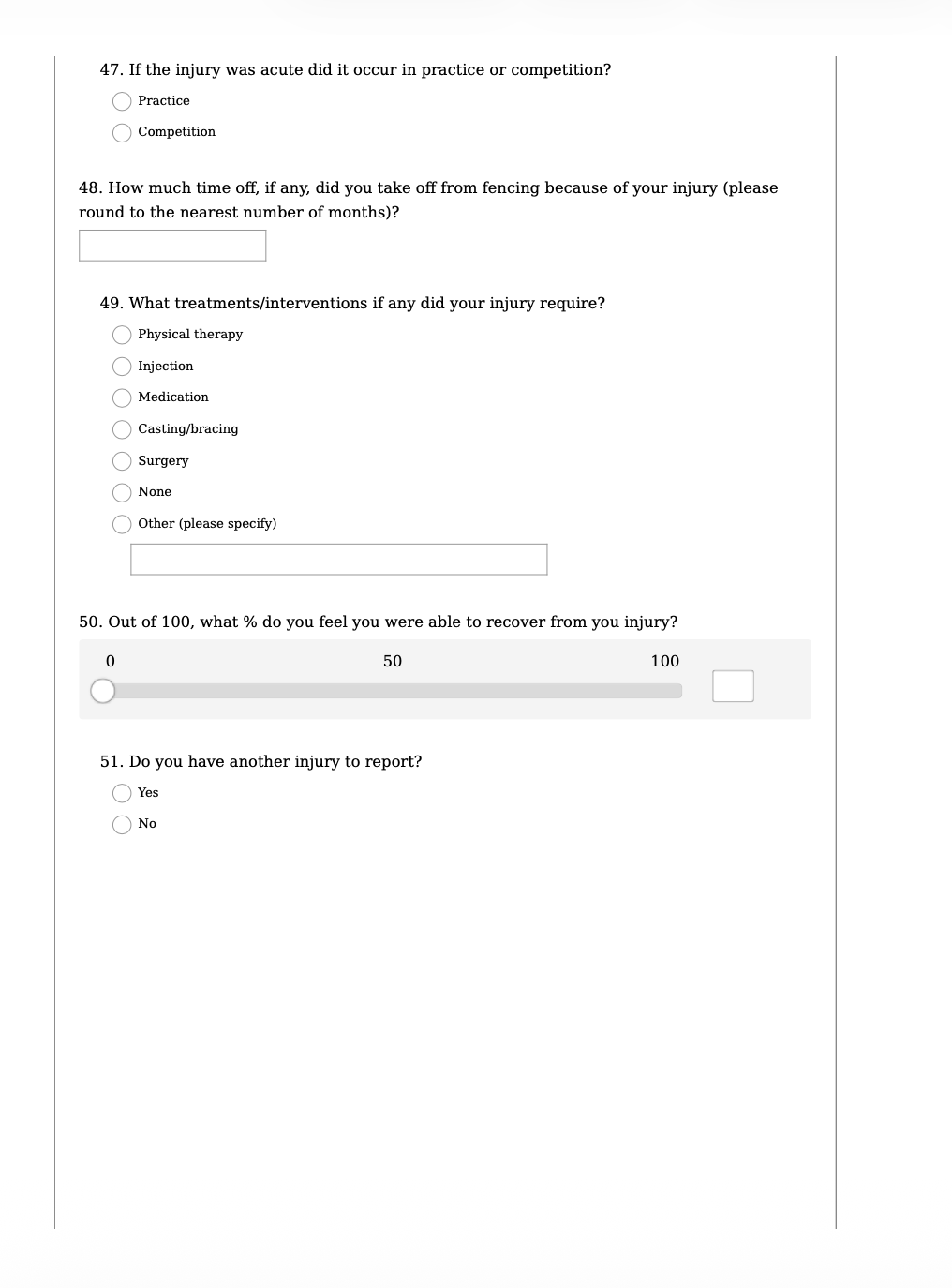


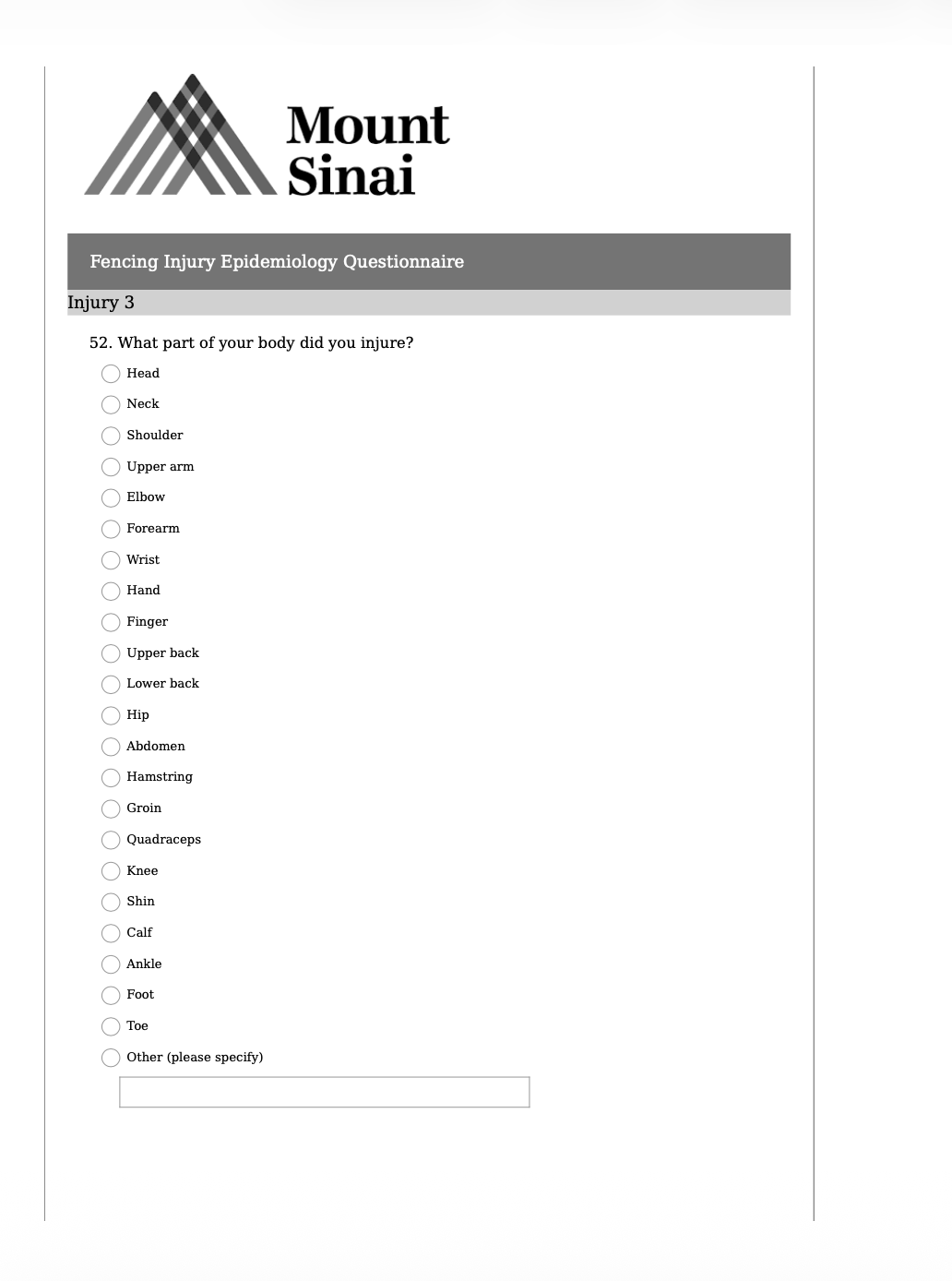


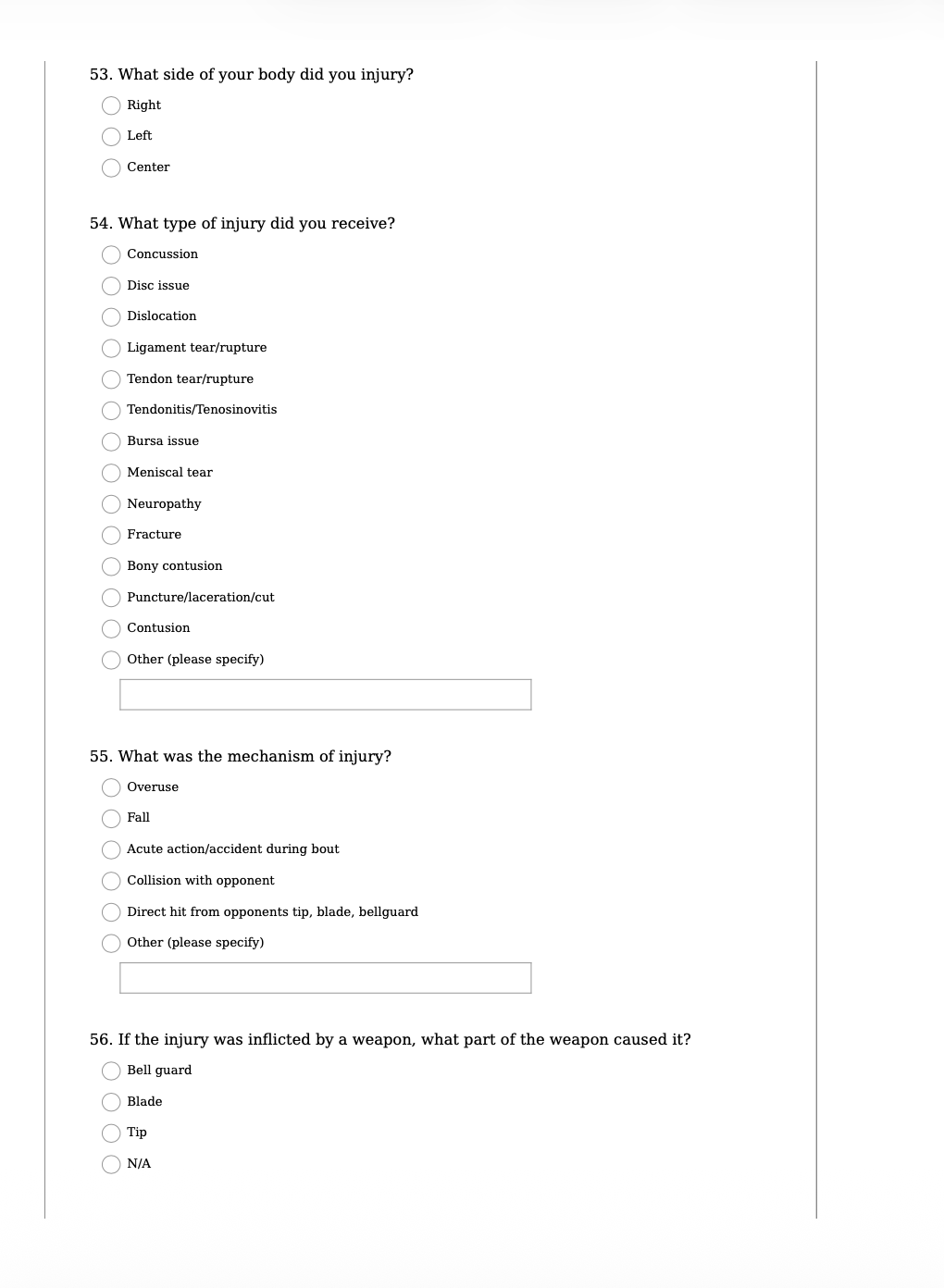


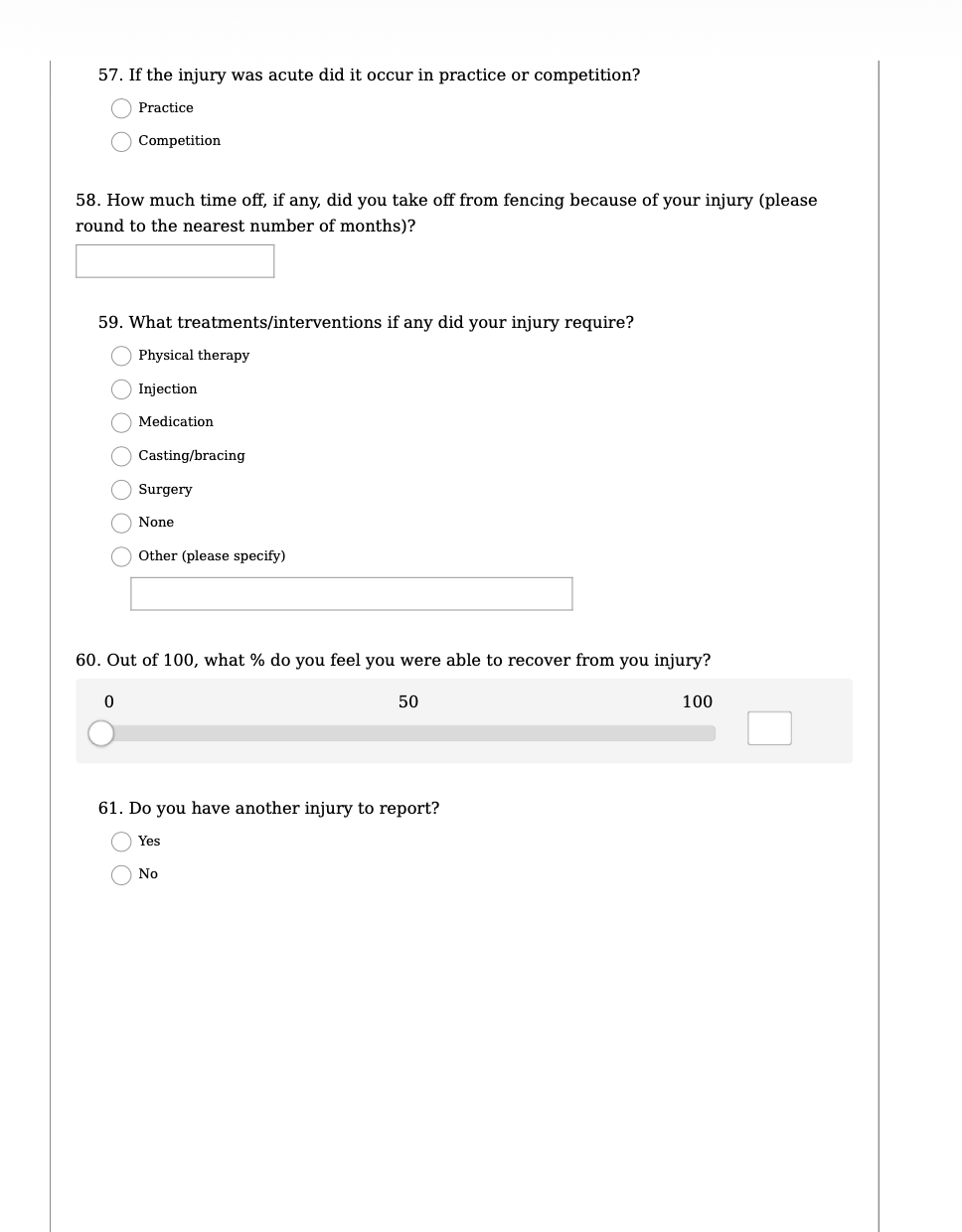


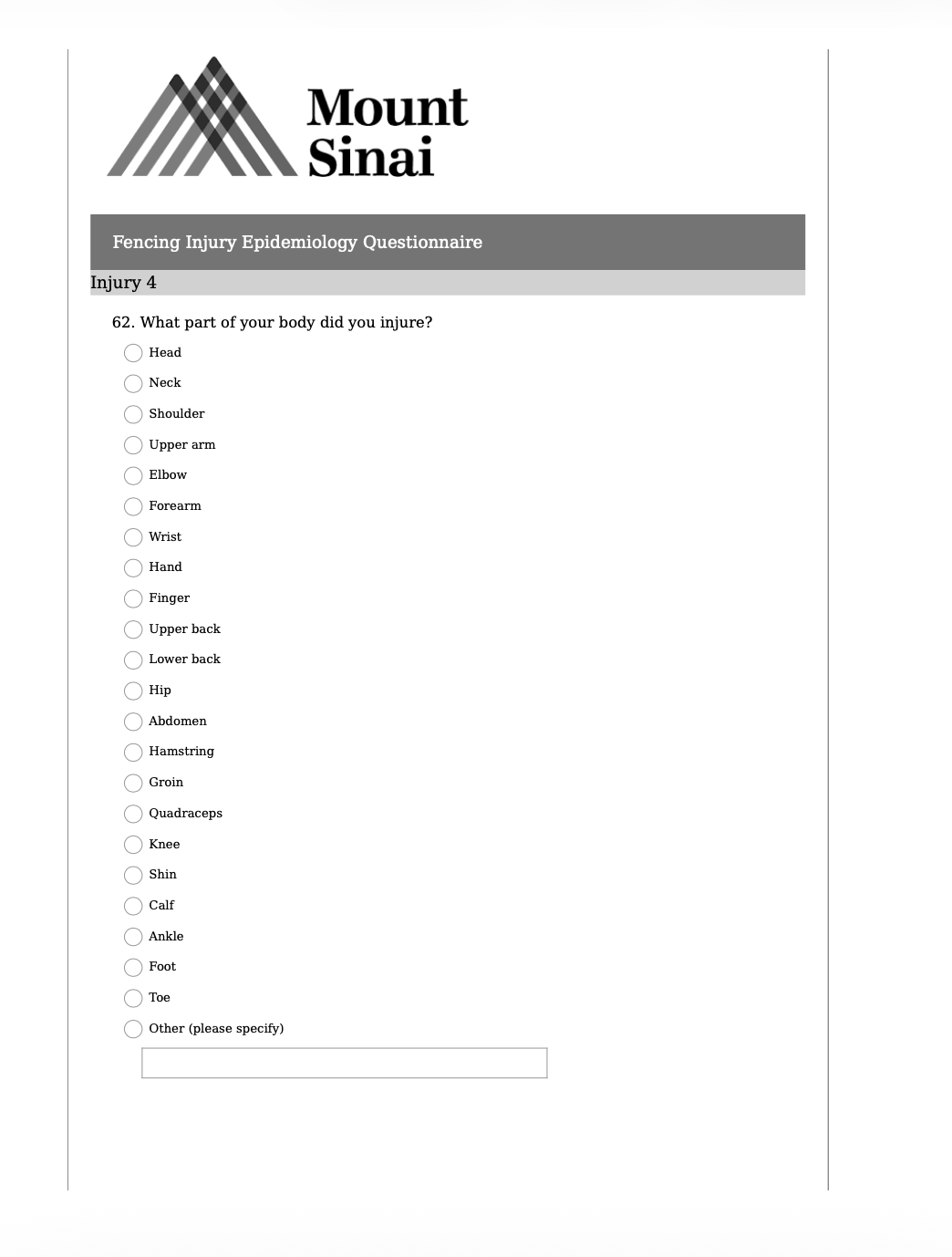


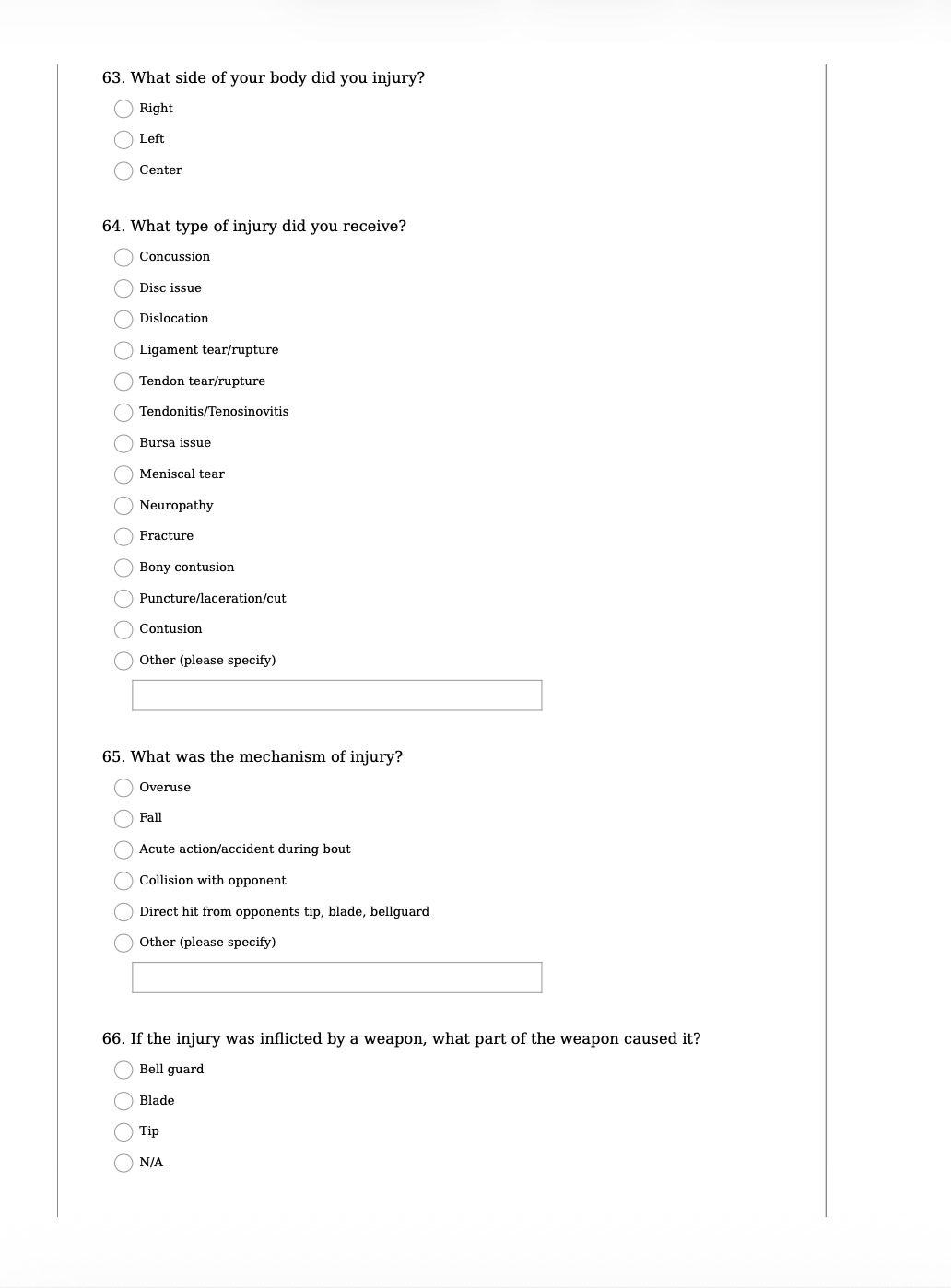


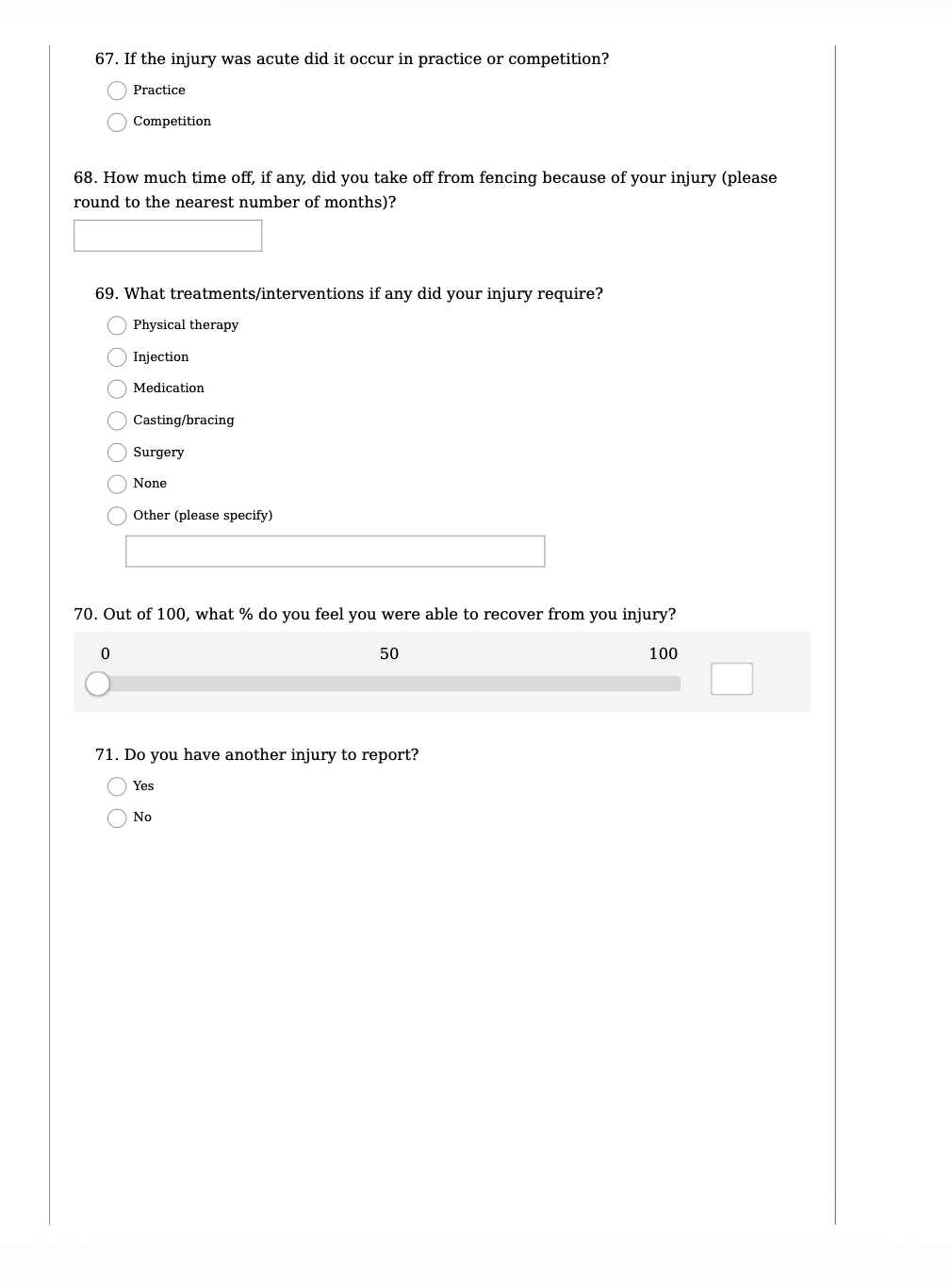


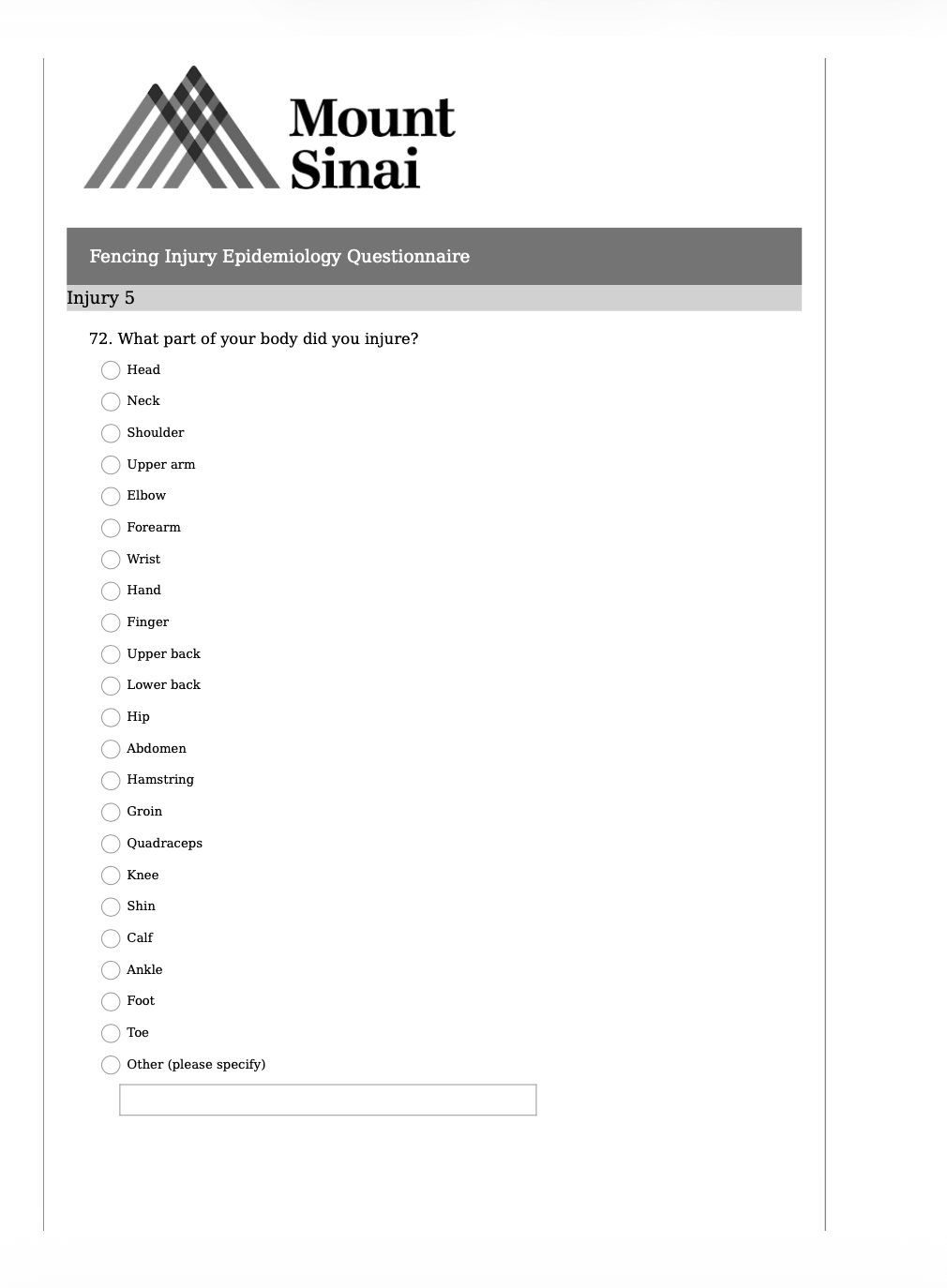


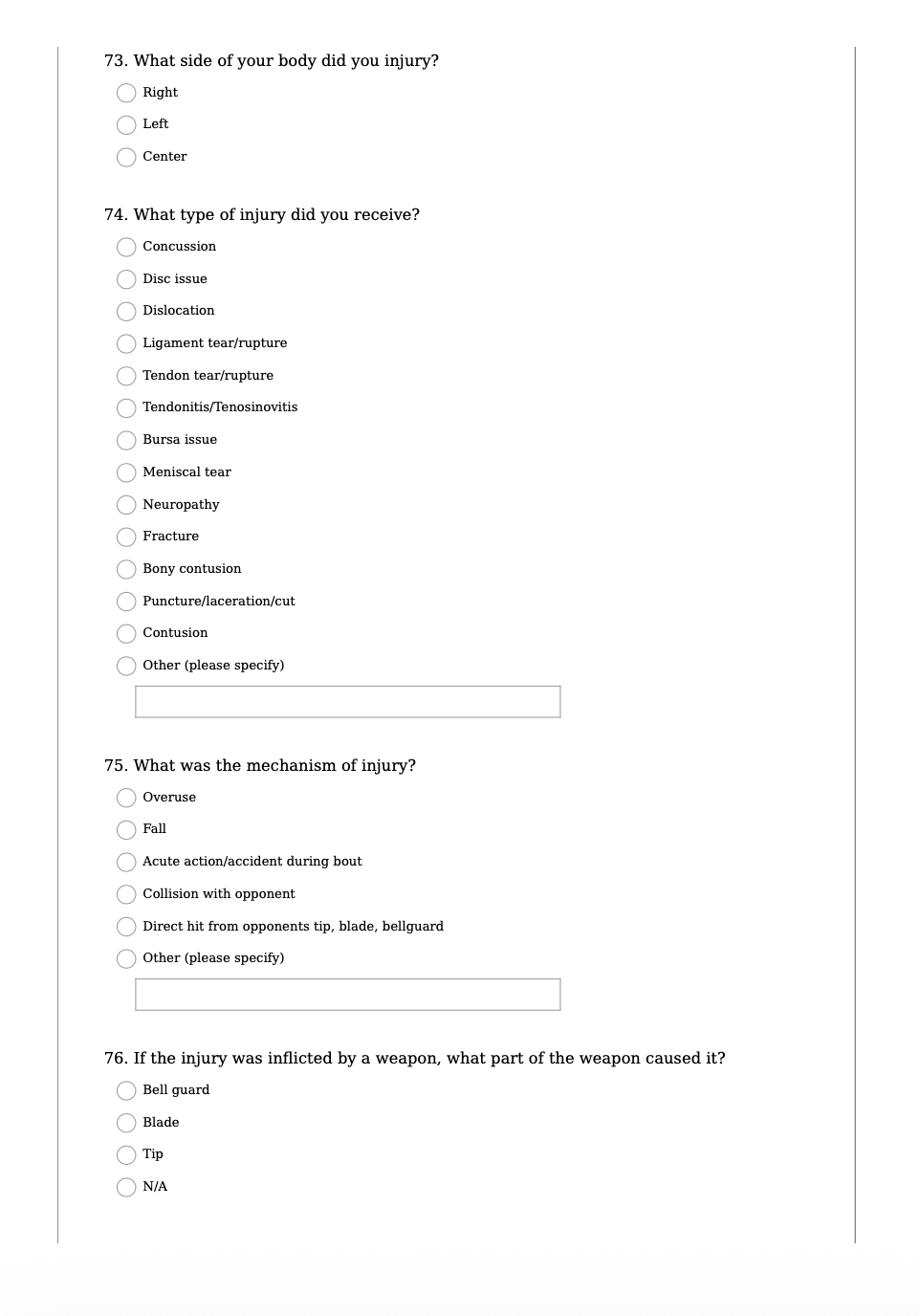


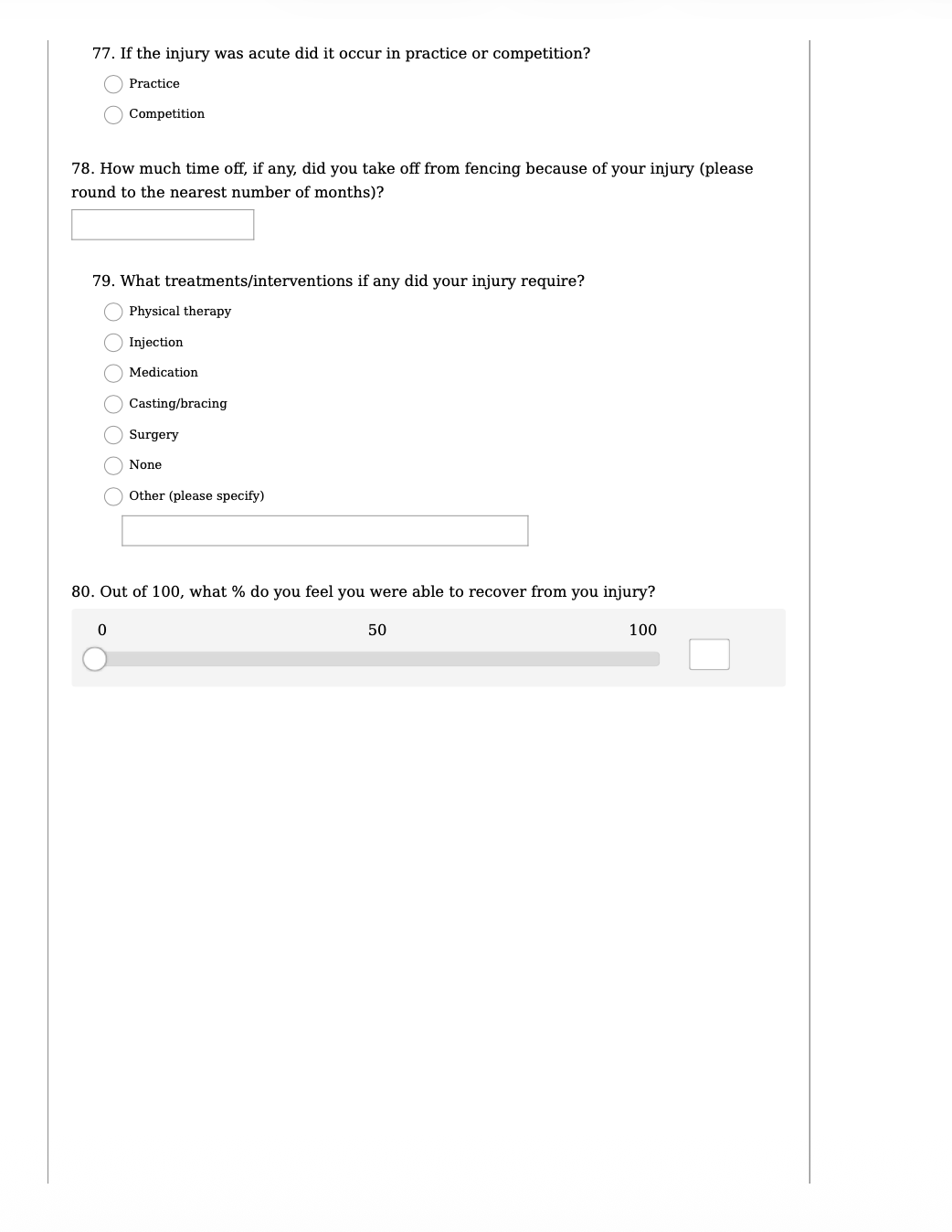


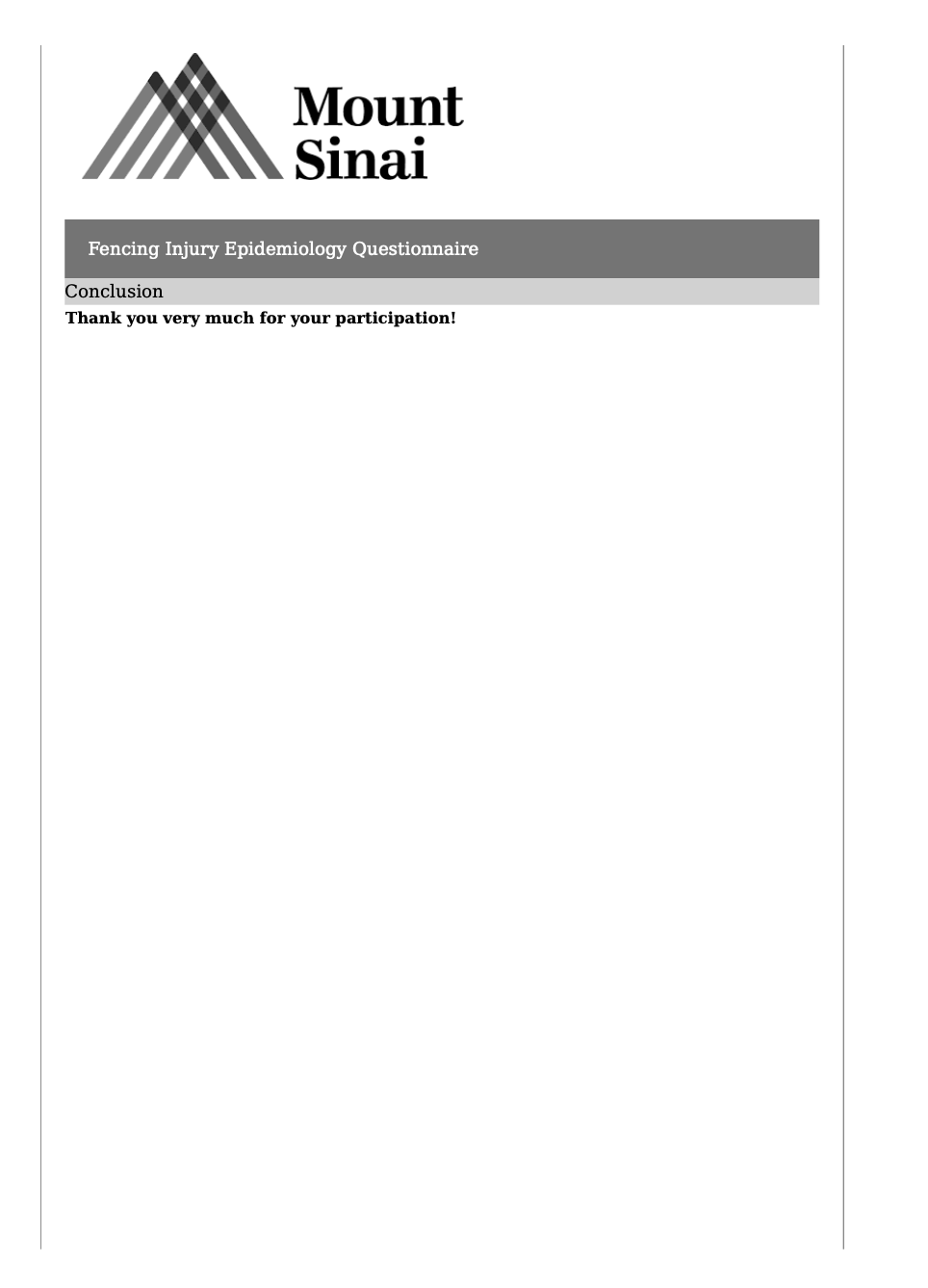


As a sensitivity analysis, we conducted additional multivariable regression analyses using categorized versions of the exposure variables to evaluate the robustness of the primary findings. These analyses were performed using both logistic regression, modeling injury burden, and Poisson regression, modeling injury count, with identical sets of categorized predictors included in each model.

For the logistic regression analysis, injury burden was defined as experiencing three or more fencing-related injuries compared with one to two injuries among injured fencers. Age at starting fencing, weekly training volume, years of fencing experience, number of competitions, and sex were included as categorical predictors (S1 Table).

For the Poisson regression analysis, the total number of fencing-related injuries was modeled as a count outcome using the same categorized predictors. Because respondents were asked to report details for only their five most severe injuries, injury counts represent a truncated measure of burden. As such, Poisson models were used for comparison purposes only and are presented as a sensitivity analysis **(S2 Table).**

Across both modeling approaches, years of fencing experience consistently demonstrated the strongest association with injury outcomes, supporting a cumulative exposure effect. Weekly training volume showed a consistent direction of association across models but was attenuated in the Poisson analysis. Age at starting fencing and sex were not independently associated with injury outcomes in either model. Overall, these supplementary analyses support the robustness of the primary spline-based logistic regression findings presented in the main manuscript.

S2 Table. Logistic regression of injury burden (≥3 injuries vs. 1–2 injuries) using categorized covariates (N=263)

| Covariates | Category | Adjusted OR | 95% CI | p-value |
| --- | --- | --- | --- | --- |
| Age at starting fencing | Early (ref) 5-15 years | 1.00 | -- | -- |
|  | Middle 16-25 years | 1.20 | 0.64–2.24 | 0.57 |
|  | Late 26-65 years | 0.99 | 0.53–1.85 | 0.97 |
| Weekly training hours | Highest (ref) 15-40 | 1.00 | -- | -- |
|  | Lowest 0-7.5 | 0.48 | 0.24–0.97 | 0.04 |
|  | Moderate 8-14 | 0.83 | 0.46–1.51 | 0.55 |
| Years of fencing | Longest (ref) 22-62 | 1.00 | -- | -- |
|  | Fewest 1-9 years | 0.35 | 0.18–0.68 | 0.002 |
|  | Moderate 10-21 years | 0.80 | 0.43–1.49 | 0.48 |
| Number of competitions | Most (ref) 9-32 | 1.00 | -- | -- |
|  | Fewest 0-4 | 0.77 | 0.42–1.41 | 0.40 |
|  | Moderate 5-8 | 1.19 | 0.58–2.41 | 0.64 |
| Sex | Female (ref) | 1.00 | -- | -- |
|  | Male | 1.47 | 0.85–2.52 | 0.17 |

Multivariable logistic regression modeling injury burden among injured fencers, defined as experiencing three or more fencing-related injuries compared with one to two injuries. Age at starting fencing, weekly training volume, years of fencing experience, number of competitions, and sex were included as categorical covariates. Results are presented as adjusted odds ratios (OR) with 95% confidence intervals. This analysis is presented as a sensitivity analysis to the primary spline-based models reported in the main manuscript.

Uninjured respondents were excluded from this analysis.

S3 Table. Poisson regression of injury count using categorized covariates (N=263)

| Covariate | Category | IRR | 95% CI | p-value |
| --- | --- | --- | --- | --- |
| Age at starting fencing | Early (ref) 5-15 years | 1.00 | -- | -- |
|  | Middle 16-25 years | 1.03 | 0.86–1.23 | 0.78 |
|  | Late 26-65 years | 1.00 | 0.84–1.20 | 0.96 |
| Weekly training hours | Highest (ref) 15-40 | 1.00 | -- | -- |
|  | Lowest 0-7.5 | 0.82 | 0.67–1.01 | 0.06 |
|  | Moderate 8-14 | 0.90 | 0.75–1.07 | 0.23 |
| Years of fencing | Longest (ref) 22-62 | 1.00 | -- | -- |
|  | Fewest 1-9 years | 0.68 | 0.56–0.83 | <0.001 |
|  | Moderate 10-21 years | 0.90 | 0.76–1.08 | 0.26 |
| Number of competitions | Most (ref) 9-32 | 1.00 | -- | -- |
|  | Fewest 0-4 | 0.96 | 0.80–1.14 | 0.66 |
|  | Moderate 5-8 | 1.00 | 0.82–1.22 | 0.97 |
| Sex | Female (ref) | 1.00 | -- | -- |
|  | Male | 1.11 | 0.95–1.26 | 0.21 |

Multivariable Poisson regression modeling the total number of fencing-related injuries using the same categorized predictors as in S1 Table. Results are presented as incidence rate ratios (IRR) with 95% confidence intervals. Because respondents were asked to report details for only their five most severe injuries, injury counts represent a truncated measure of burden. This analysis is presented for comparison purposes only.

Uninjured respondents were excluded from this analysis
