# Supplementary material for: Injury patterns and cumulative injury burden among U.S. competitive fencers: A survey
Source: PLoS One. 2026 Mar 16;21(3):e0344263. doi: 10.1371/journal.pone.0344263 (PMC12991207; doi:10.1371/journal.pone.0344263)
Supplement: S2 Table — Multivariable Poisson regression modeling the total number of fencing-related injuries using the same categorized predictors as in S1 Table. Results are presented as incidence rate ratios (IRR) with 95% confidence intervals. Because respondents were asked to report details for only their five most severe injuries, injury counts represent a truncated measure of burden. This analysis is presented for comparison purposes only. Uninjured respondents were excluded from this analysis. (DOCX) [file pone.0344263.s003.docx]

S2 Table. Poisson regression of injury count using categorized covariates (N=263)

| Covariate | Category | IRR | 95% CI | p-value |
| --- | --- | --- | --- | --- |
| Age at starting fencing | Early (ref) 5-15 years | 1.00 | -- | -- |
|  | Middle 16-25 years | 1.03 | 0.86–1.23 | 0.78 |
|  | Late 26-65 years | 1.00 | 0.84–1.20 | 0.96 |
| Weekly training hours | Highest (ref) 15-40 | 1.00 | -- | -- |
|  | Lowest 0-7.5 | 0.82 | 0.67–1.01 | 0.06 |
|  | Moderate 8-14 | 0.90 | 0.75–1.07 | 0.23 |
| Years of fencing | Longest (ref) 22-62 | 1.00 | -- | -- |
|  | Fewest 1-9 years | 0.68 | 0.56–0.83 | <0.001 |
|  | Moderate 10-21 years | 0.90 | 0.76–1.08 | 0.26 |
| Number of competitions | Most (ref) 9-32 | 1.00 | -- | -- |
|  | Fewest 0-4 | 0.96 | 0.80–1.14 | 0.66 |
|  | Moderate 5-8 | 1.00 | 0.82–1.22 | 0.97 |
| Sex | Female (ref) | 1.00 | -- | -- |
|  | Male | 1.11 | 0.95–1.26 | 0.21 |

Multivariable Poisson regression modeling the total number of fencing-related injuries using the same categorized predictors as in S1 Table. Results are presented as incidence rate ratios (IRR) with 95% confidence intervals. Because respondents were asked to report details for only their five most severe injuries, injury counts represent a truncated measure of burden. This analysis is presented for comparison purposes only.

Uninjured respondents were excluded from this analysis.
